# HIV-1 Nef Changes the Proteome of T Cells Extracellular Vesicles Depleting IFITMs and Other Antiviral Factors

**DOI:** 10.1016/j.mcpro.2023.100676

**Published:** 2023-11-07

**Authors:** Mara E. da Silva-Januário, Cristina S. da Costa, Lucas A. Tavares, Ana K. Oliveira, Yunan C. Januário, Andreia N. de Carvalho, Murilo H.A. Cassiano, Roger L. Rodrigues, Michael E. Miller, Soledad Palameta, Clarice W. Arns, Eurico Arruda, Adriana F. Paes Leme, Luis L.P. daSilva

**Affiliations:** 1 Centro de Pesquisa em Virologia (CPV) and Departamento de Biologia Celular e Molecular, Faculdade de Medicina de Ribeirão Preto, Universidade de São Paulo, Ribeirão Preto, São Paulo, Brazil; 2 Laboratório Nacional de Biociências (LNBio), Centro Nacional de Pesquisa em Energia e Materiais (CNPEM), Campinas, São Paulo, Brazil; 3 Instituto de Biologia, Universidade de Campinas (UNICAMP), Campinas, São Paulo, Brazil

**Keywords:** Extracellular vesicles, EVs, exosomes, HIV, Nef, IFITMs, antiviral activity

## Abstract

Extracellular vesicles (EVs) are biomolecule carriers for intercellular communication in health and disease. Nef is a HIV virulence factor that is released from cells within EVs and is present in plasma EVs of HIV-1 infected individuals. We performed a quantitative proteomic analysis to fully characterize the Nef-induced changes in protein composition of T cell-derived EVs and identify novel host targets of HIV. Several proteins with well-described roles in infection or not previously associated with HIV pathogenesis were specifically modulated by Nef in EVs. Among the downregulated proteins are the interferon-induced transmembrane 1, 2, and 3 (IFITM1-3) proteins, broad-spectrum antiviral factors known to be cell-to-cell transferable by EVs. We demonstrate that Nef depletes IFITM1-3 from EVs by excluding these proteins from the plasma membrane and lipid rafts, which are sites of EVs biogenesis in T cells. Our data establish Nef as a modulator of EVs' global protein content and as an HIV factor that antagonizes IFITMs.

## Highlights

• Nef is an HIV-1 virulence factor released in extracellular vesicles (EVs).
• EVs may deliver either proviral or antiviral factors to cells changing infection.
• The study shows that Nef causes global alterations in the proteomics of T cell EVs.
• Nef removes antiviral interferon-induced transmembrane proteins (IFITMs) from EVs.

## Graphical Abstract

**Figure:**
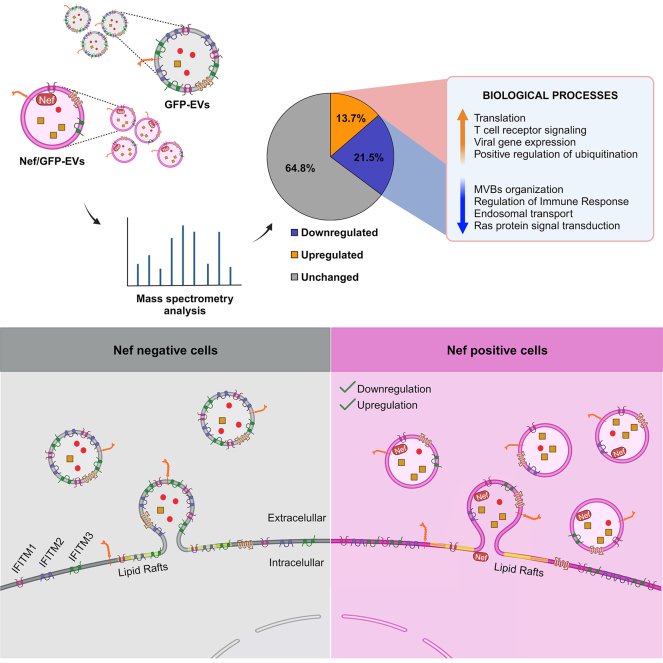

## In Brief

Extracellular vesicles (EVs) released from cells may carry either proviral or antiviral cargo to other cells. Nef, an HIV-1 virulence factor, is released in EVs from infected cells. We discovered that Nef causes global changes in the protein makeup of EVs derived from T cells, which may affect their overall function. Notably, Nef removes antiviral interferon-induced transmembrane proteins from EVs. These findings shed light on how HIV-1 manipulates host cells and provides new insights into potential targets for therapeutic interventions.

Cells actively release extracellular vesicles (EVs) that carry an extensive repertoire of molecules involved in intercellular communication (1, 2). An important function of EVs is the cell-to-cell transference of proteins involved in antiviral defense, antagonizing infection by several viruses, such as the HIV (3, 4). On the other hand, viruses counteract EVs by modifying their content and favoring infections (3, 4).

The HIV accessory protein Nef is a major regulator of intracellular protein trafficking and alters several host proteins' subcellular distribution (5, 6), making the cellular environment more suitable for virus replication and spread. Some of the Nef targets include the HIV-1 receptor CD4 (7), major histocompatibility complex-I molecules (8), and SERINC3/5 (9, 10), cell intrinsic antiviral restriction factors.

Another mechanism used by Nef to promote HIV infection is modifying the release and content of EVs (4). HIV and simian immunodeficiency viruses Nef proteins are released from cells within EVs (11, 12, 13, 14, 15). The EV-mediated uptake of Nef was shown to trigger proapoptotic signaling cascades in bystander CD4^+^ T cells (11) and to impair the cholesterol metabolism in macrophages, leading to proinflammatory responses due to the reorganization of inflammatory receptors in lipid raft domains (16). Nef removes CD4 present in the surface of EVs released by T cells, which otherwise impair HIV infection *in vitro* (17). HIV-1 infection was shown to induce relocation of cargo among different subpopulations of EVs from T cells. While some proteins became incorporated into virions, SERINC3 was shown to move away from virions into nonviral EVs in a Nef-dependent manner (18). Finally, Nef-positive EVs carry mRNA, microRNAs (19, 20), and proteins (21, 22) that contribute to viral pathogeneses.

Despite the described role of Nef in inducing EVs secretion and changing specific host proteins' levels in these vesicles, no global analysis was reported to define the Nef-mediated alterations in EVs protein content broadly. In this study, we demonstrated that Nef produces unprecedented changes in the population of proteins secreted in EVs and identified novel host targets of Nef. By performing a quantitative proteomic analysis of vesicles released by T cells expressing Nef, we found that several proteins involved in immune response are modulated. Among the downregulated proteins were three members of the interferon-induced transmembrane (IFITM) protein family (namely, IFITM1, 2, and 3), which are involved in antiviral response against several viruses, such as Zika (23), dengue (24), influenza (24) and HIV (25, 26).

IFITMs perturb HIV-1 infection *via* at least three reported mechanisms: virus entry impairment (25), reduction of viral proteins synthesis (27), and infectivity decrease (26). IFITMs traffics through the endosomal pathway and their subcellular localization correlates to antiviral function (28). We show that Nef reduces the surface levels of IFITMs and their association with lipid raft domains, which are the main sites of EVs biogenesis and HIV entry in T cells. We also show that EVs mediate the transfer of IFITM3 to bystander T cells, suggesting that Nef prevents IFITM3 uptake by uninfected T cells inhibiting IFITM3 loading into EVs released by infected/donor cells. Thus, the findings described here provide the proteomic mapping of the global changes triggered by Nef in the protein content of T cell EVs and indicates that Nef is an HIV-1 factor that antagonizes antiviral IFITMs.

## Experimental Procedures

### Cell Culture

The following cell lines were obtained through the NIH HIV Reagent Program, Division of AIDS, NIAID, NIH: A3.01 CD4^+^ T cells, originally deposited by Thomas Folks (29); MT4 T cells originally deposited by Dr Douglas Richman (30, 31); and TZM-bl cells, contributed by Dr John C. Kappes, Dr Xiaoyun Wu, and Tranzyme Inc (32). Suspension cells were grown in RPMI 1640 medium (Thermo Fisher Scientific) supplemented with 100 U/ml penicillin, 0.1 mg/ml streptomycin, 2 mM L-glutamine, and 10% fetal bovine serum (FBS) (Thermo Fisher Scientific). PEAK cells, which are HEK293 cells transfected with the large T antigen of SV-40 (33) were kindly provided by Dr Reuben Siraganian (National Institutes of Health). HEK293T and HeLa (CCL2) cells were obtained from American Type Culture Collection. Adherent cells were grown in Dulbecco's modified Eagle's medium (DMEM) (Thermo Fisher Scientific), supplemented as described above. All cells were cultured at 37 °C with 5% CO_2._ After acquisition, cells were immediately expanded, frozen as aliquots and stored in vapor phase liquid nitrogen. Cell aliquots were used up to the 15th passage and discarded. PCR tests for *mycoplasma* were negative.

### Expression Vector and Reagents

The pMSCV-internal ribosome entry site (IRES)-GFP and pMSCV-Nef-IRES-GFP retroviral vectors were previously described (17). To generate a pMSCV-IRES-GFP based vector expressing the primary Nef allele NA7, isolated from a human HIV-1 infected individual (34), we PCR amplified the DNA fragment encoding this Nef variant from the pNefNA7.IRES2.eGFP plasmid (35), and subcloned it into pMSCV-IRES-GFP using the BglII and SalI restriction sites. The full-length open reading frames of human IFITM1, IFITM2, and IFITM3 were amplified by PCR with oligonucleotides that introduced a double hemagglutinin (HA) tag in frame with the C terminus. Amplified IFITMs-2xHA sequences were then inserted as EcoRI/NotI fragments into the multicloning site of the pCI-neo expression vector (Promega), resulting in the following plasmids: pCI-neo IFITM1-2xHA, pCI-neo IFITM2-2xHA, and pCI-neo IFITM3-2xHA. For the pcDNA5/FRT/TO_IFITM3-2xHA construct the IFITM3-2xHA sequence was inserted as an EcoRI/HindIII fragment into the multicloning site of pcDNA5/FRT/TO (Thermo Fisher Scientific). Transfections were performed using Lipofectamine 2000 reagent (Catalog no. 11668019; Thermo Fisher Scientific), according to the manufacturer’s protocol.

### Doxycycline Inducible IFITM3 Cell Line Generation

The construct pcDNA5/FRT/TO_IFITM3-2xHA was transfected in T-REX 293 cells (Thermo Fisher Scientific). At 48 h post transfection, stable transfected cells were selected in fresh medium containing 5 μg/ml of blasticidin (Invitrogen) and 200 μg/ml hygromycin B (Invitrogen). After, the selected clones were pooled and expanded in selective medium. We confirmed the doxycycline inducible IFITM3-2xHA expression by Western blot with an anti-HA antibody and used these cells to produce and isolated EVs containing IFITM3-2xHA.

### Antibodies

The table below describes the commercial antibodies used, their catalog numbers, and their company.

Western blot and immunofluorescence antibodies.

**Table:** 

Anti-Alix (sc-49267)	(Santa Cruz Biotechnology)
Anti-β-actin (MA1-91399)	(Thermo Fisher Scientific)
Anti-β-actin (sc-47778)	(Santa Cruz Biotechnology)
Anti-CD4 (2009-09)	(Novocastra)
Anti-CD63 (sc-15363)	(Santa Cruz Biotechnology)
Anti-EEA1 (610456)	(BD Biosciences)
Anti-Flotillin1 (ab41927)	(Abcan)
Anti-GAPDH (G9545)	(Sigma-Aldrich)
Anti-GFP	Gift from R. Hedge (MRC)
Anti-ΗΑ (H3663)	(Sigma-Aldrich)
Anti-HLA-A (15240-1-AP)	(Proteintech)
Anti-IFITM1 (60074-1-Ig)	(Proteintech)
Anti-IFITM2 (66137-1-Ig)	(Proteintech)
Anti-IFITM3/2 (11714-1-AP)	(Proteintech)
Anti-Lat1 (sc-54229)	(Santa Cruz Biotechnology)
Anti-TfR (136800)	(Thermo Fisher Scientific)
Anti-Tsg101 (976126)	(BD Bioscencies)
Anti-Nef (2949)	(NIH AIDS Reagent Program)
Anti-Neurexin1 (ab77596)	(BD Bioscencies)
Anti-Syntenin1 (ab133267)	(Abcan)
Anti-IgG Mouse-HRP	(GE)
Anti-IgG Rabbit-HRP	(GE)
Anti-IgG Goat-HRP	(GE)
Anti-IgG Mouse-Alexa 594	(Thermo Fisher Scientific)
Anti-IgG Rabbit-Alexa 647	(Thermo Fisher Scientific)

### Transduction of T Cells for the Expression of GFP, Nef NL4/GFP, or Nef NA7/GFP

Retroviruses encoding GFP alone or Nef alleles and GFP were generated by cotransfection of PEAK cells in a 100 mm tissue culture dish with 3 μg pVSV-G, 6 μg pCL-Eco, and 9 μg of either pMSCV-IRES-GFP, pMSCV-Nef NL4-IRES-GFP, or pMSCV-Nef NA7-IRES-GFP plasmids, using 30 μl of 25 kDa linear PEI (1 mg/ml stock solution) transfection reagent (Polysciences Inc). Cell supernatants containing retroviruses were collected 36 h after transfection. A3.01 cells were incubated with the supernatants for 24 h. Transduced cells were washed with PBS and cultivated in complete medium for 72 h. GFP-positive cells were sorted by FACSAria III (BD). After sorting, A3.01 cells expressing Nef NL4 and GFP (Nef/GFP), Nef NA7 and GFP (Nef NA7/GFP), or GFP alone (GFP) were expanded in culture, and homogeneous GFP expression was confirmed by flow cytometric analysis and Western blot prior to use in experiments. These cells are excellent tools to study CD4 and major histocompatibility complex-I downregulation by Nef (17, 36, 37).

### Extracellular Vesicles and Total Lysates

These experiments were performed following the minimal information for studies of extracellular vesicles (MISEV2018) guidelines (38). A3.01 cells (GFP, Nef/GFP and Nef NA7/GFP) were washed with PBS and reincubated with RPMI containing 10% of the commercially available “exosome-depleted FBS” (Thermo Fisher Scientific, cat # A2720801) or FBS-EVs-depleted RPMI prepared in-house (as described in the Preparation of FBS-EVs-depleted media section) at 6.0 × 10^5^ cells/ml confluence. Cell-conditioned media (200 ml of each sample) was collected 48 h later. A preliminary test assay was performed within 24, 48, and 72 h of cell culture to set the best time for EV production, with cells reaching the confluence of approximately 1 × 10^6^ cells/ml, 1.3 × 10^6^ cells/ml and 1.6 × 10^6^ cells/ml at 24, 48, and 72 h of culture, respectively. EVs enrichment in the preparations were performed using an initial three-step sequential centrifugation at 4 °C: 10 min at 300*g*, to remove cells; 10 min at 2000*g*, to remove dead cells; and 30 min at 10,000*g*, to remove cell debris (39). The supernatant was passed through a 0.22 μm filter and ultracentrifuged at 100,000*g* for 3 h and 30 min at 4 °C followed by one wash (suspension in PBS/centrifugation at 100,000*g*) to remove residual soluble serum and secreted proteins (Rotor: AH-629-36 mL; K factor: 242). EVs preparations were then concentrated for 1 h and 10 min at 100,000*g*, 4 °C using a tabletop ultracentrifuge (Rotor: TLA-110-5.1 mL; K factor: 13).

To obtain EVs from HEK IFITM3-2xHA cells, after 24 h of seeding cells were washed with PBS and incubated in DMEM supplemented with 10% of commercially available exosome-free FBS (Thermo Fisher Scientific) or FBS-EVs-depleted DMEM prepared in-house (as described in the Preparation of FBS-EVs-depleted media section), and doxycycline (Sigma-Aldrich) to induce IFITM3-2xHA expression. After additional 36 h of incubation, conditioned media were collected and processed for EVs isolation as described for A3.01 cells. The ultracentrifugations were performed at 100,000*g* for 1 h 30 at 4 °C in an ultracentrifuge (Thermo Fisher Scientific), followed by a wash (suspension in PBS/centrifugation at 100,000*g*) to remove residual soluble serum and secreted proteins (Rotor: T-1250-23 mL Fixed; K-factor: 68.7). The final pellets of EVs from A3.01 cells (GFP, Nef/GFP and Nef NA7/GFP) and HEK IFITM3-2xHA cells were re-suspended in 50 μl of Dulbecco's phosphate-buffered saline (DPBS) and freshly analyzed. For the mass spectrometry (MS) analysis, the final pellets of EVs from A3.01 cells (GFP and Nef/GFP) were frozen at −80 °C after ultracentrifugation for posterior digestion (described in the Trypsin digestion of extracellular vesicle proteins section). The corresponding cells were washed in cold PBS (∼1.0 × 10^7^ cells), centrifuged at 300*g* for 5 min at 4 °C and then re-suspended in 200 μl of lysis buffer (50 mM Tris–HCl [pH 7.5], 150 mM NaCl, 10% [vol/vol] glycerol, 5 mM EDTA, 1% [vol/vol] Triton X-100) supplemented with protease inhibitor cocktail (Sigma-Aldrich). Lysates were clarified by centrifugation at 16,000*g* for 20 min at 4 °C, mixed with sample buffer, and boiled.

### Production FBS-EVs-Depleted Media

To remove the EVs from FBS in culture media, a standard previously described protocol was used (38, 39). Briefly, 250 ml of RPMI or DMEM media containing 20% of FBS were ultracentrifuged at 100,000*g* for 18 h at 4 °C (Rotor: AH-629-36 mL; K factor: 242). Afterward, the supernatant was transferred to new tubes, mixed with 100 U/ml penicillin, 0.1 mg/ml streptomycin, 2 mM L-glutamine, and 250 ml of RPMI or DMEM, and then filtered with Stericup Sterile Vacuum Filtration Systems (0.22 μm). The FBS-EVs-depleted media was used in all the experiments, with exception of the EVs preparation for MS analysis, in which was used commercially available exosome-depleted FBS from Thermo Fisher Scientific.

### Scanning Electron Microscopy

EVs enrichment from 23 ml of culture supernatants of 1.0 × 10^7^ A3.01 Nef NL4-3/GFP or GFP cells (48 h cell-conditioned media) were pelleted as described above and the pellet was resuspended in 1 ml of Fixation Buffer [2% glutaraldehyde and 2% paraformaldehyde in cacodylate buffer (0.1 M cacodylate pH 7.4 containing 0.025% of CaCl_2_)] and incubated for 2 h at 4 °C. The pellets were then washed by two rounds of ultracentrifugation at 100,000*g* for 2 h at 4 °C (Rotor: TH-660; K factor: 44.4) in 4 ml of cacodylate buffer. Then, the samples were resuspended in 100 μl of cacodylate buffer and were incubated overnight at 4 °C. After that, the 100 μl sample were placed on 10-mm round coverslips previously treated with Biobond (Electron Microscopy Sciences) and allowed to adhere for 30 min at room temperature. The coverslips were incubated with 1% osmium tetroxide in cacodylate buffer for 2 h at room temperature. After five washes for 3 min with Milli-q water, the coverslips were incubated overnight at 4 °C in Milli-q water. Afterward, the coverslips were incubated with thiocarbohydrazide solution (0.5 g of thiocarbohydrazide diluted in 25 ml Milli-q water and then filtered with MF-Millipore Membrane Filter/0.22 μm pore size) for 10 min at room temperature and then washed for five times for 3 min with Milli-q water. Next, the coverslips were incubated with 1% osmium tetroxide in cacodylate buffer for 30 min at room temperature and then washed five times for 3 min with Milli-q water. This was followed by incubation in thiocarbohydrazide solution for 10 min at room temperature and five times washes for 3 min with Milli-q water. Next, the coverslips were incubated again with 1% osmium tetroxide in cacodylate buffer for 30 min at room temperature and then washed five times for 3 min with Milli-q water. Then, the coverslips were submitted to a series of ethanol incubations (once with 30% for 5 min, 50% for 5 min, 70% for 5 min, 90% for 5 min, 95% for 5 min, and twice with 100% for 5 min) and critically point-dried with liquid CO_2_ in a Leica EM CPD300 critical-point dryer (Leica Microsystems). The coverslips were mounted on aluminum stubs with charcoal glue (EM Sciences) and coated with gold in a Bal-Tec SCD 050 Sputter Coater (BAL-TEC). The samples were examined with the JEOL JSM- 6610LV scanning electron microscope (JEOL) available at Electron Microscopy Multiuser Laboratory at Department of Cell and Molecular Biology (FMRP-USP).

### Nanoparticle Tracking Analysis

EVs suspended in DPBS were filtered through a 0.22 μm filter, at room temperature, and vesicles concentration and size were determined using nanoparticle tracking analysis (NTA) (NanoSight appliance NS300, Malvern Instruments) with NTA 3.2 Dev Build 3.2.16. The capture settings were as follows: camera setting: sCMOS; laser type: green; slider shutter: 1300; slider gain: 512; frames per second: 25.0; number of frames: 749; temperature: 22.6 to 22.7 °C; viscosity: 0.937 to 0.939 cP. Additionally, the camera level was set up to >14 at which all particles were distinctly visible and the threshold was determined to particles within an ideal 20 to 100 particles/frame range.

### EVs Transference to Acceptor Cells (MT4 and TZM-bl)

After the production and enrichment, EVs from the HEK IFITM3-2xHA cells were quantified by NTA and incubated at different concentrations, and for different amounts of times with the acceptor cells, depending on the assay performed. For Western blot transfer analysis, the EVs were incubated for 3 h with acceptor cells (MT4 T cells or TZM-bl). For immunofluorescence transfer analysis, EVs were labeled with PKH26 reagent (Sigma-Aldrich), according to the manufacturer’s instructions, and then incubated for 1 and 2 h with TZM-bl and 1 h with MT4. Briefly, 950 μl of Diluent C were added to 50 μl of freshly isolated EVs or DPBS (as a control without EVs), and 6 μl of PKH26 were added to each sample. Samples were mixed for 30 s by gentle pipetting, followed by incubation for 5 min at room temperature. Next, for quenching, 2 ml 10% bovine serum albumin in DPBS were added to each sample, mixed, the volume was brought to 15.5 ml with DPBS and added on top of 1.5 ml of a 0.971 M sucrose solution in a 17 ml ultracentrifuge tube. The EVs were pelleted by ultracentrifugation at 100,000*g* for 3 h and 30 min at 4 °C (Rotor: AH-629-17 mL; K factor: 284). The EVs pellet were re-suspended in 50 μl of DPBS, quantified by NTA, and incubated with the acceptor cells for the times stated in the figure legends. The PBS controls, without EVs, followed the same procedure that the samples with EVs.

### Surface Biotinylation Assay

A3.01 cells (GFP and Nef/GFP) at the confluence of 1.0 × 10^7^ were washed with cold PBS and incubated with 0.2 mg/ml of EZ-Link Sulfo-NHS-LC-Biotin (Thermo Fisher Scientific) in cold PBS for 30 min at 4 °C. Cells were washed with cold PBS twice and incubated with 50 mM NH_4_Cl for 5 min at 4 °C and then washed with cold PBS three times. Cells collected by centrifugation at 200*g* for 5 min at 4 °C and treated with lysis buffer, containing protease inhibitor cocktail (Sigma-Aldrich), for 20 min at 4 °C. The lysates were centrifuged at 16,000*g* for 20 min at 4 °C to obtain the supernatants. Proteins in the supernatants were quantified using BioRad Protein Assay reagent (BioRad), equalized and incubated with 30 μl of prewashed NeutrAvidin Plus UltraLink Resin (Thermo Fisher Scientific) for 3 h in an orbital shaker at 4 °C. Beads were washed four times with 1 ml of cold lysis buffer and proteins eluted from beads with 30 μl of sample buffer were compared to 1% of the total protein applied to the beads by Western blot analysis.

### Enrichment of Lipid Rafts

A3.01 cells (5.0 × 10^7^ of GFP or Nef/GFP cells) were lysed in 1 ml of ice-cold TNE buffer (10 mM Tris–HCl, pH 7.5, 150 mM NaCl, 5 mM EDTA) containing 0.5% Triton X-100, and protease inhibitor cocktail (Sigma-Aldrich). Lysates were passed through a 21-gauge needle 20 times and centrifuged at 1000*g* for 10 min at 4 °C. A volume of 1 ml of the clarified supernatant fraction was mixed with 4 ml of 85% sucrose in TNE buffer and layered on the bottom of a polyallomer 15 ml centrifuge tube. The lysate was overlaid with 5 ml of 35% sucrose in TNE buffer, and them with 5 ml of 5% sucrose in TNE buffer. The tubes were ultracentrifuged in an AH-629-17 mL rotor at 155,846*g* for 24 h at 4 °C. Nine fractions of 1.6 ml were collected from the top of the gradient and proteins were precipitated with 15% trichloroacetic acid (JT Baker). Precipitated proteins were washed with cold acetone, dried at room temperature and submitted to a 14% SDS PAGE and Western blot analysis.

### Western Blot Analysis

Protein extracts were heat-denaturated in sample buffer and proteins were separated by 10% or 14% SDS-PAGE under reducing conditions and electro-transferred to nitrocellulose membranes (Millipore). Membranes were stained with Ponceau S (Sigma-Aldrich) and immunoblotted with the indicated antibodies. Proteins were detected by using a homemade enhanced chemiluminescence solution (solution 1: 1 M Tris–HCl [pH 8.5], 250 mM luminol, 90 mM p-coumaric acid; and solution 2: 30% H2O2, 1 M Tris-HCl [pH 8.5]). Proteins were visualized by the ChemiDoc Imaging System equipped with the ImageLab (https://fiji.sc/) software (Bio-Rad Laboratories). Signals were quantified by densitometry scanning, using ImageJ.

### Immunofluorescence

A3.01 T cells expressing GFP or Nef/GFP and MT4 T cells were seeded on 13-mm-diameter coverslips pretreated with Poly-L-lysine solution (Sigma-Aldrich), fixed and processed for immunofluorescence assay as previously described (36). HeLa and TZM-bl cells were seeded on 13-mm-diameter coverslips. The next day cells were transfected and after 16 h transfection cells were fixed, permeabilized, and processed for immunofluorescence assay as described previously (36). Cells were analyzed on a Zeiss confocal laser-scanning microscope (LSM) 780 (Zeiss). Postacquisition image processing was performed using ImageJ (https://fiji.sc/).

### Cell Viability and Flow Cytometry Analysis

HeLa cells were co-transfected with pCIneo-IFITM3-2xHA and pIRES-GFP or pNef NL4-IRES-GFP plasmids by using Lipofectamine 2000 transfection reagent. At 20 h posttransfection, unfixed cells were incubated at 4 °C with 3% PBS-bovine serum albumin blocking solution followed by mouse monoclonal anti-HA antibody (Catalog no. H3663; Sigma-Aldrich) and F(ab')2-goat anti-mouse IgG(L + H) cross-absorved Alexa Fluor 647 secondary antibody (Catalog no. A21237; Thermo Fisher Scientific). Then, the cells were fixed with 4% paraformaldehyde and maintained in PBS. Flow cytometry data were acquired with a Diva flow cytometer (BD Biosciences) and analyzed using FlowJo (https://www.flowjo.com/) software. Cell viability was determined by flow cytometric analysis using the BD Horizon Fixable Viability Stain 575V (FVS575V). The FVS575V working solution was prepared by diluting 1 μl of FBS575V stock solution [200 μg of FVS575V in 340 μl of fresh cell culture-grade dimethyl sulfoxide (DMSO; Sigma-Aldrich D2650)] in 1 ml of PBS. Cells (GFP, Nef NL4-3/GFP, and Nef NA7/GFP) were cultured in FBS-EVs-depleted RPMI media (described above) for 24, 48, and 72 h. Then, 1.0 × 10^7^ cells for each condition were washed two times with ice-cold PBS, spun at 300*g* for 5 min at 4 °C, and incubated with 100 μl of ice-cold FVS575V working solution for 15 min at 4 °C. Next, cells were washed two times with ice-cold PBS, centrifuged at 300*g* for 5 min at 4 °C, and fixed with 4% paraformaldehyde in ice-cold PBS. The cell samples were analyzed in a FACSymphony A5 Cell Analyzer (BD Biosciences) at the Ribeirão Preto Center for Cell-Based Therapy/Hemotherapy. As gate strategy we first selected cells using side scatter and forward scatter parameters, and then selected singlets and then live cells (negative for FVS575V). We used the FloJo (https://www.flowjo.com/) software for data analyses.

### Trypsin Digestion of Extracellular Vesicle Proteins

The frozen EV pellet was suspended in 8 M urea, reduced with 5 mM DTT for 25 min at 56 °C and alkylated with 14 mM iodoacetamide for 30 min at room temperature and light protected. The alkylation reaction was quenched with 5 mM DTT for 15 min at room temperature. After dilution of urea to 1.6 M with a solution containing 50 mM ammonium bicarbonate and 1 mM calcium chloride, 1 μg trypsin was added per sample and incubated for 16 h at 37 °C. The reaction was stopped with 0.4% formic acid, and peptides were desalted with C18 stage tips (40), dried in a vacuum concentrator, reconstituted in 0.1% formic acid and stored at −20 °C for subsequent analysis by liquid chromatography coupled with tandem mass spectrometry (41).

### MS Analysis

GFP-EVs and Nef/GFP-EVs derived peptides were loaded into the EASY-nLC II (Proxeon Biosystem) coupled to the mass spectrometer LTQ Orbitrap Velos (Thermo Fisher Scientific). The peptides were eluted with a gradient of 2 to 30% acetonitrile in 0.1% formic acid over 120 min at a flow rate of 300 nl/min, using the analytical column PicoFrit C18 (20 cm × 75 μm id, 5 μm; New Objective). The peptides were analyzed by the mass spectrometer operating in the positive mode for data dependent acquisition. Precursor ions (300–1600 m/z) were scanned in the Orbitrap with resolution defined to r = 60.000 and 1.0 × 10^6^ target ions. Up to 20 most intense ions were isolated through a 3 m/z window and activated by collision induced dissociation. Dynamic exclusion was enabled with an exclusion list of up to 500 ions, an exclusion duration of 60 s and a count repetition of 1.

### Proteomic Data Analysis

The raw files were processed using the MaxQuant v.1.5.8 (https://www.maxquant.org/) using the Andromeda search engine (42, 43) against the UniProt Human Protein Database concatenated with Bovin Protein Database, enhanced GFP sequence from Human-cytomegalovirus and Nef protein sequence from HIV-1 virus proteome (released in December 2017, with 98.579 entries). The maximum allowed mass tolerance was set to 20 ppm for the precursor in the main search, 6 ppm in the second search, and 0.5 Da of mass tolerance for fragment ions. Enzyme specificity was set to trypsin with a maximum of two missed cleavages. Carbamidomethylation of cysteine (+57.02146 Da) was set as a fixed modification, oxidation of methionine (+15.99491 Da) and protein N-terminal acetylation (+42.01056 Da) were set as variable modifications. The false discovery rate at the peptide and protein levels were both set to 0.01. A minimal ratio count of 1 and a 2-min window for matching between runs was required for quantitation. Proteoforms were automatically merged in a single protein group, except when identified by at least one unique peptide. Protein identifications assigned as “Reverse”, “Contaminant”, and “Bovine exclusive sequence” were excluded from further analysis.

The following analysis was performed using Perseus v.1.4.2 (44) available in the MaxQuant environment (https://www.maxquant.org/perseus/, accessed in December 2021). The reverse, contaminants proteins and exclusively bovine proteins were excluded. For further analysis, we considered proteins exclusive to humans and those shared between human and bovine taxonomies.

The label-free quantitation (LFQ) intensity values were considered for quantitative protein data and were obtained for each EVs sample. For this analysis, the LFQ data were log2 transformed, and the imputation of missing values was carried out for missing values, which were replaced by random numbers that are drawn from a normal distribution, representing low abundance measurements. The statistical analysis was performed using Student's *t* test (*p*-value < 0.05) and Log2 Fold Change (log2 ratio of Nef/GFP LFQ intensity) of the three independent samples of GFP-EVs (N = 3) and Nef/GFP-EVs (N = 3). Tables containing all data analyzed are available in Supplemental Information. For data visualization, volcano plot and hierarchical clustering analysis with z-score values of log2 LFQ intensities were performed in the R environment using the Euclidian distance and single linkage method. The data were submitted to the Vesiclepedia database (45) and analyzed using FunRich (https://www.funrich.org/) software 3.1.3 (46, 47). The data were also searched for biological processes in the Gene Ontology (GO) database, using Enrichr (48, 49). Vesiclepedia and GO database were accessed on July, 2020.

### Alignment

Different proteins sequences from human IFITM1 (Gene ID: 8519; Reference Sequence: NP_003632.4), IFITM2 (Gene ID: 10581; Reference Sequence: NP_006426.2) and IFITM3 (GeneID: 10410; Reference Sequence: NP_066362.2) were retrieved from NCBI. The sequences were alignment using the free software Clustal Omega alignment (50).

### Statistical Analysis

All statistical data are shown as mean ± SEM and the number of samples “n” is indicated in the legend of each experiment. The different statistical tests used are described in the legends of the respective figures. The *p*-values are labeled as follows: ∗ represents *p* < 0.05; ∗∗ represents *p* < 0.01; ∗∗∗ represents *p* < 0.001. Differences were considered statistically significant for *p* < 0.05. Data were plotted on graphs and analyzed using R statistical (https://www.r-project.org/) software or GraphPad Prism 5.0 software (https://www.graphpad.com/).

### Experimental Design and Statistical Rationale

We performed a quantitative proteomic analysis of the Nef-induced changes in the protein composition of T cell-derived EVs (GFP-EVs and Nef/GFP-EVs). MS-based proteomics sequencing was run in biological triplicates for EVs from GFP-EVs (control) and Nef/GFP-EVs to capture biological variation. The MS analysis was carried out using the label-free method in MaxQuant software (42, 43) and Perseus (44), both accessed in December 2012. For this analysis, the following were excluded: 16 reverse sequences, 100 contaminants proteins, and 99 exclusive bovine protein sequences. For statistical analysis, we used the 818 identified exclusive human proteins, and 345 proteins shared between human and bovine taxonomies. The log2 intensities from the proteomics analysis are normally distributed and a two-sided Student's *t* test was used in Perseus v. 1.3.0.4 software to determine proteins, differentially abundant between GFP-EVS and Nef/GFP-EVS (*p*-value ≤ 0.05). To avoid reducing the number of differentially abundant molecules and failing to perform process enrichment and the multiomics analysis, we considered a nonadjusted *p*-value obtained from Student's *t* test. (i) imputation of missing values was carried out for missing values, (ii) carrying out a comprehensive search of EVs and HIV markers using public databases. Hierarchical cluster heat map, volcano plot, and biological processes/pathways enrichment using FunRich and GO database/Enrichr (41, 42, 43, 44) were used to characterize the group of molecules associated with the Nef-EVs.

## Results

### Quantitative Proteomics Maps Nef-Induced Changes in Protein Profile of T Cells EVs

**Fig. 1:**
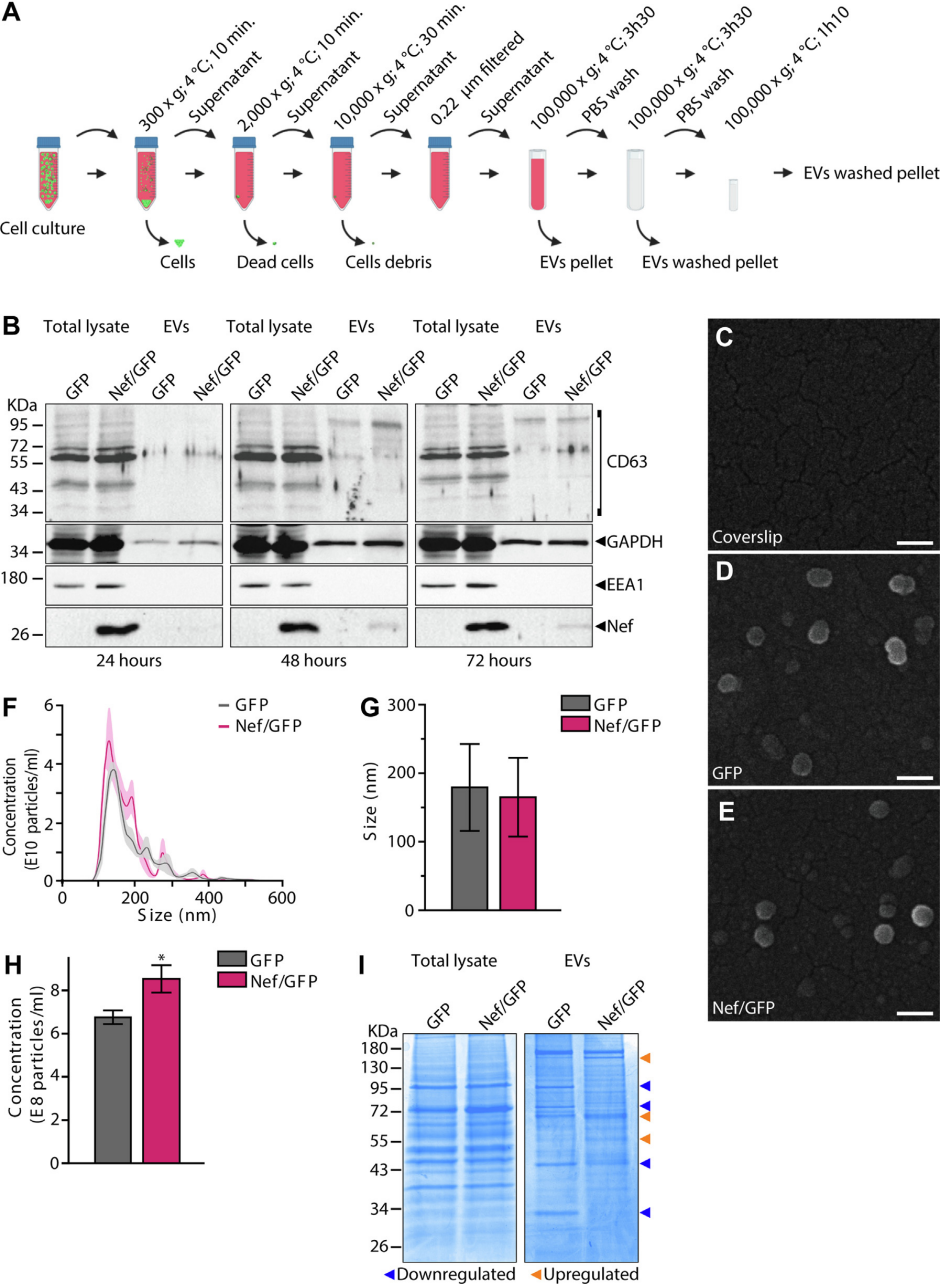
**Characterization of EVs produced by A3.01 GFP and Nef/GFP T cells.***A*, experimental design. Culture supernatants were subjected to three-step sequential centrifugation at 4 °C: 10 min at 300*g*, to remove cells; 10 min at 2000*g*, to remove dead cells; and 30 min at 10,000*g* to remove cellular debris. Then, the supernatant was passed through a 0.22 μm filter. The filtrate was ultracentrifuged at 100,000*g* for 3 h 30 at 4 °C in an ultracentrifuge, followed by washing (suspension in PBS/centrifugation at 100,000*g*) to remove residual soluble serum and secreted proteins. The supernatant was discarded and the pellet containing the EVs was resuspended in PBS and subjected to an additional ultracentrifugation step for 1 h and 10 min at 100,000*g*, 4 °C using a bench top ultracentrifuge (details in Experimental Procedures). The final pellet was used immediately for different assays. *B*, A3.01 GFP and Nef/GFP T cells were incubated with an EV-depleted medium for 24, 48, and 72 h at 37 °C. Then the supernatant was collected and the EVs were isolated by differential ultracentrifugation as described in (*A*) and the cells were lysed for analysis of protein levels by Western blot. *C*–*E*, SEM analysis of EVs released from A3.01 GFP and Nef/GFP T cells. A corresponding image of the treated coverslip with no sample, using the same settings, is provided as reference (*C*). The scale bar represnts 0.2 μm. *D*, GFP-EVs. *E*, Nef/GFP-EVs. *F*, representative graph of an analysis by NTA from n = 3 biological replicates, with five technical runs. The line is the average value, and the shadow indicates ±standard error of the mean (SEM). *G*–*H*, the diameter and concentration of the EVs were characterized by NTA. The bar graph represents the mean ± SEM of EVs size and concentration. Student's *t* test; ∗*p* < 0.05. *I*, total protein content of cells and EVs samples visualized by SDS-PAGE stained with Coomassie blue. The band profile observed was partially distinct for GFP-EVs and Nef/GFP-EVs samples as highlighted by *arrowheads*. EV, extracellular vesicles; NTA, nanoparticle tracking analysis.

**Supplemental Figure 1:**
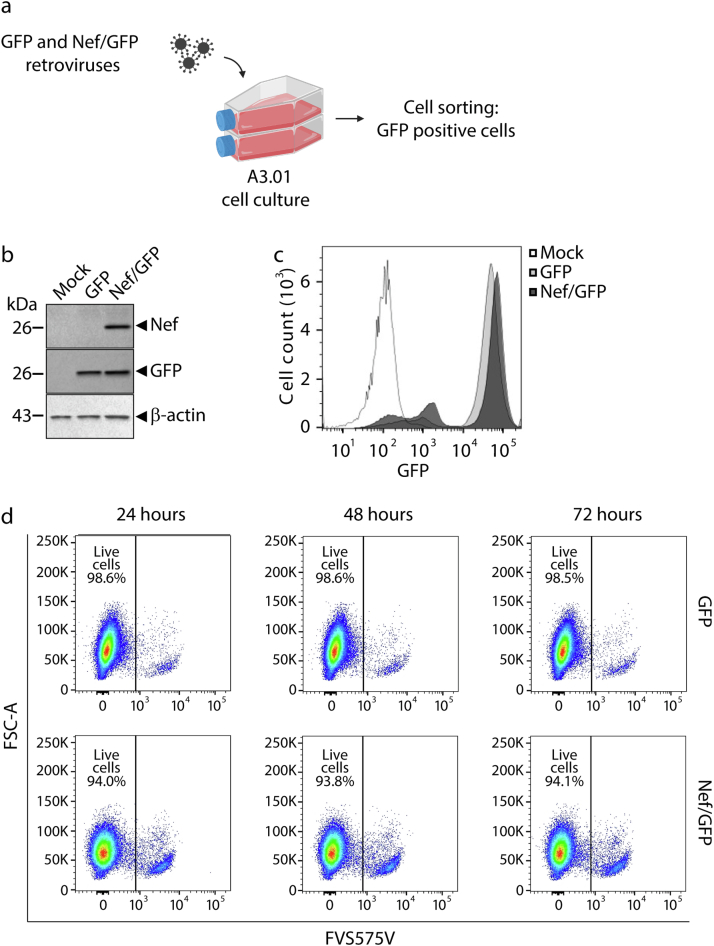


A3.01 T cells were transduced with bicistronic IRES-based retroviral vectors to express both HIV-1 NL4 Nef and GFP (Nef/GFP) or GFP alone (GFP) and selected by cell sorting (supplemental Fig. S1). Culture supernatants of Nef/GFP and GFP cells conditioned during 24, 48, or 72 h were processed by differential ultracentrifugation (Fig. 1*A*) to obtain EV-enriched fractions, which were analyzed by Western blot. EV fractions obtained from 48 h conditioned supernatants were used in future analysis, as no relative increase of EV marker proteins was noticeable after this time point (Fig. 1*B*), and without compromising cell viability (supplemental Fig. S1*D*). Scanning electron microscopy (Fig. 1, *C*–*E*) and NTA confirmed the presence of EVs in the samples, with approximately 170 nm of diameter in average (Fig. 1, *F*–*G*). NTA also indicated an increased concentration of EVs released by Nef/GFP cells compared with GFP cells, as previously reported for other cell types (11, 51) (Fig. 1, *F* and *H*). Interestingly, SDS-PAGE analysis of total lysates and EVs lysates from Nef/GFP and GFP cells suggested differences in EV protein profile (Fig. 1*I*).

**Fig. 2:**
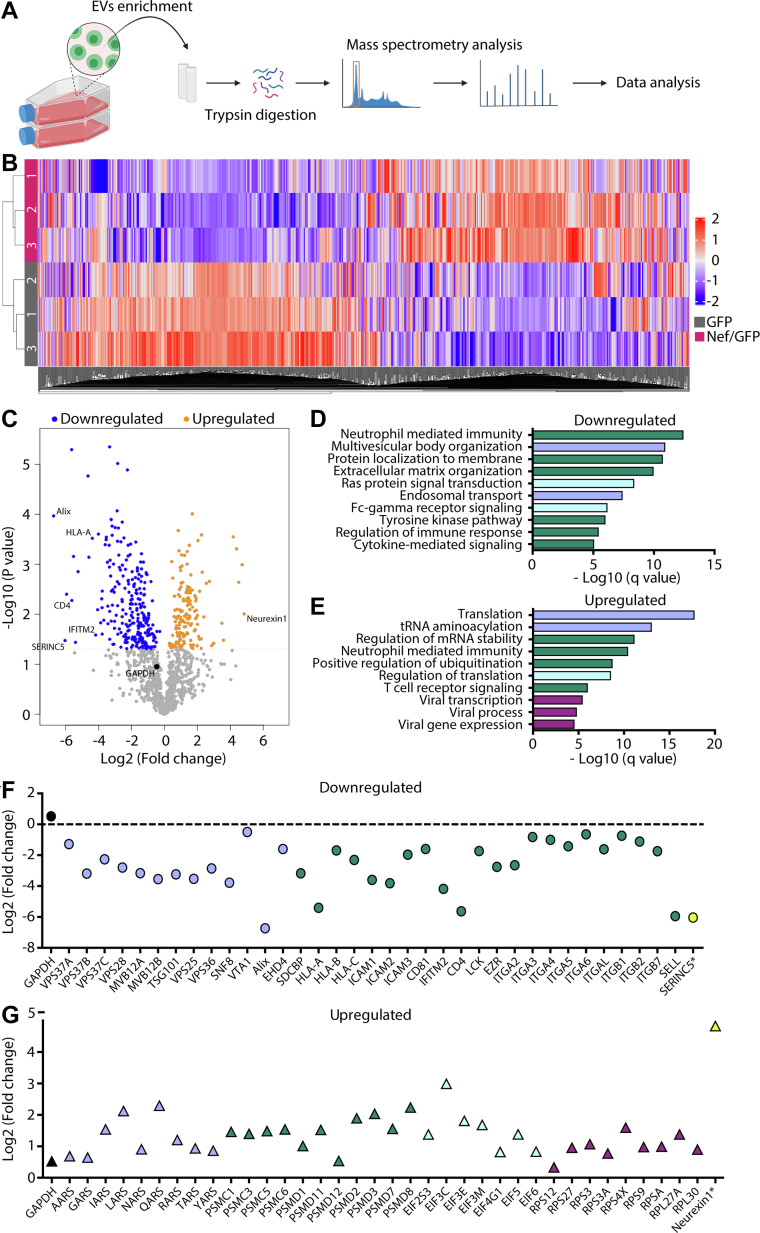
**Quantitative proteomic analysis showing that HIV-1 Nef changes the protein profile of T cells EVs.***A*, experimental design. After EVs enrichment, proteins were extracted from the pellets of the GFP-EVs and Nef/GFP-EVs, and digestion with trypsin was performed. The peptide mixture was desalted and analyzed by liquid chromatography coupled with tandem mass spectrometry (LC-MS/MS). *B*, hierarchical clustering analysis with z-scored log2 LFQ intensity values of all proteins identified in the GFP-EVs triplicates (*gray*) and Nef/GFP-EVs triplicates (*Burgundy*). Values for each protein (*columns*) in each sample (*rows*) are colored based on the protein’s relative abundance, in which high (*orange*) and low (*blue*) are indicated based in the color scale bar shown in the *right side* of the figure. *C*, volcano plot of statistically significant proteins (−log10 *p*-value; Student's *t* test) against fold change (log2 ratio of Nef/GFP-EVs/GFP-EVs LFQ intensity) of proteins levels in Nef EVs. *Blue* and *orange dots* represent proteins that are significantly (*p*-value < 0.05) downregulated or upregulated in Nef/GFP-EVs, respectively. *Gray dots* are unchanged proteins (*p*-value > 0.05) and the *black dot* represent GAPDH. Relevant proteins are indicated. *D* and *E*, Gene Ontology biological process terms with adjusted *p*-values <0.05 enriched in downregulated (*D*) and upregulated (*E*) proteins. *F* and *G*, the panels show examples of significantly (Student's *t* test; *p*-value < 0.05) downregulated (*F*) and upregulated proteins (*G*) (log2 ratio of Nef/GFP-EVs/GFP-EVs LFQ intensity) in response to Nef expression. Different colors are linked to the biological process shown in (*D* and *E*). ∗Two proteins that are not related to (*D* and *E*) but were strongly downregulated (SERINC5) and upregulated (Neurexin1) are shown as reference in *yellow*. GAPDH abundance is shown in *black dot* with *p*-value >0.05. EV, extracellular vesicles; LFQ, label-free quantitation.

**Supplemental Figure 2:**
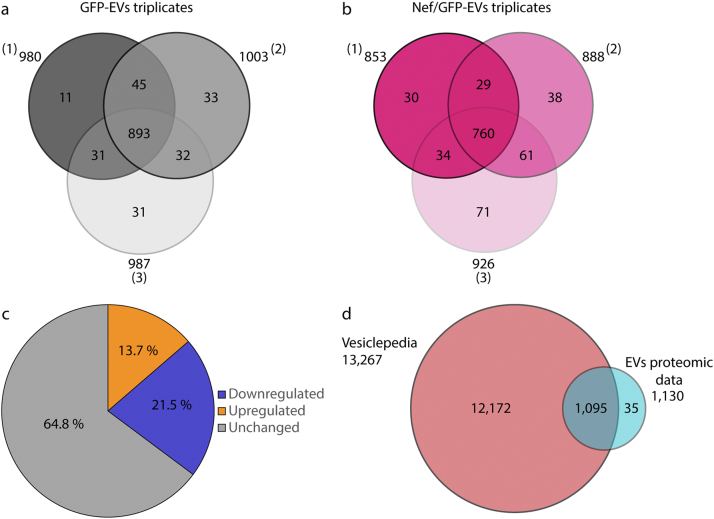


The proteomes of EVs from GFP (GFP-EVs) and Nef/GFP (Nef/GFP-EVs) cells were analyzed using quantitative mass spectrometry and LFQ intensity to compare the relative abundance of the proteins (Fig. 2*A*). The proteomic analysis allowed the quantitation of several proteins in GFP-EVs and Nef/GFP-EVs triplicate samples (supplemental Table S1). A total of 1076 proteins were identified in GFP-EVs and 1023 in Nef/GFP-EVs (supplemental Fig. S2, *A* and *B* and supplemental Table S1). The filtered dataset of the GFP-EVs and Nef/GFP-EVs was subjected to statistical analysis using Student’s *t* test (*p*-value < 0.05), which resulted in 401 proteins with differential abundances, and applying Benjamini-Hochberg correction (Adjusted *p*-value < 0.05) 166 proteins showed significant differential abundance (supplemental Table S2). Of all proteins observed from Nef/GFP and GFP EVs proteomics, 64.8% showed no alteration in abundance, while 21.5% had the abundance levels reduced and 13.7% increased in the presence of Nef expression (supplemental Fig. S2*C*). Among the proteins identified, 3.1% (35 proteins) had not been previously found in EVs according to the proteomics database Vesiclepedia (supplemental Fig. S2*D* and supplemental Table S3).

Unsupervised hierarchical clustering analysis grouped the biological triplicates of GFP-EVs and Nef/GFP-EVs proteomes and revealed the protein diversity in these EVs and a clear variation in protein levels as a consequence of Nef expression (Fig. 2*B*). The proteins were plotted in a Volcano plot, and some proteins of interest were highlighted (Fig. 2*C*). Our positive controls of Nef activity, CD4 and HLA-A, were downregulated in Nef/GFP-EVs, as previously reported (Fig. 2*C*) (17). Notably, the anti-HIV proteins IFITM2 and SERINC5 were also downregulated by Nef in EVs, at similar intensities as CD4 and HLA-A (Fig. 2*C*). Neurexin1 represents a protein with increased levels in EVs due to Nef expression (Fig. 2*C*). Biological pathways enrichment of proteins downregulated or upregulated in EVs showed that Nef actively modulates their protein components (Fig. 2, *D* and *E* and supplemental Table S4). Intriguingly, several proteins belonging to the endosomal sorting complex required for transport (ESCRT) machinery were downregulated in Nef/GFP-EVs, such as Alix, Tsg101, and VPS37 isoforms, as well proteins involved in innate immune response and extracellular matrix organization (Fig. 2, *D* and *F*). In contrast, proteins involved in mRNA stability, protein translation, and ubiquitination regulation were more abundant in EVs due to Nef expression (Fig. 2, *E* and *G*).

### Nef Reduces the Levels of Specific EV Markers and Host Defense Proteins in EVs

The proteomic data revealed several host proteins known to affect the HIV replication cycle to be present in T cell EVs. Some of them were present in differential levels in Nef/GFP-EVs, such as CD4 (7), HLA-A (8), EHD4 (52), Ezrin (52), and IFITM2 (25) (Fig. 2, *C* and *F*). One of the most differentially abundant protein was IFITM2, which had its levels reduced by approximately 4-fold (Log2) in Nef/GFP-EVs (Fig. 2, *C* and *F*).

**Fig. 3:**
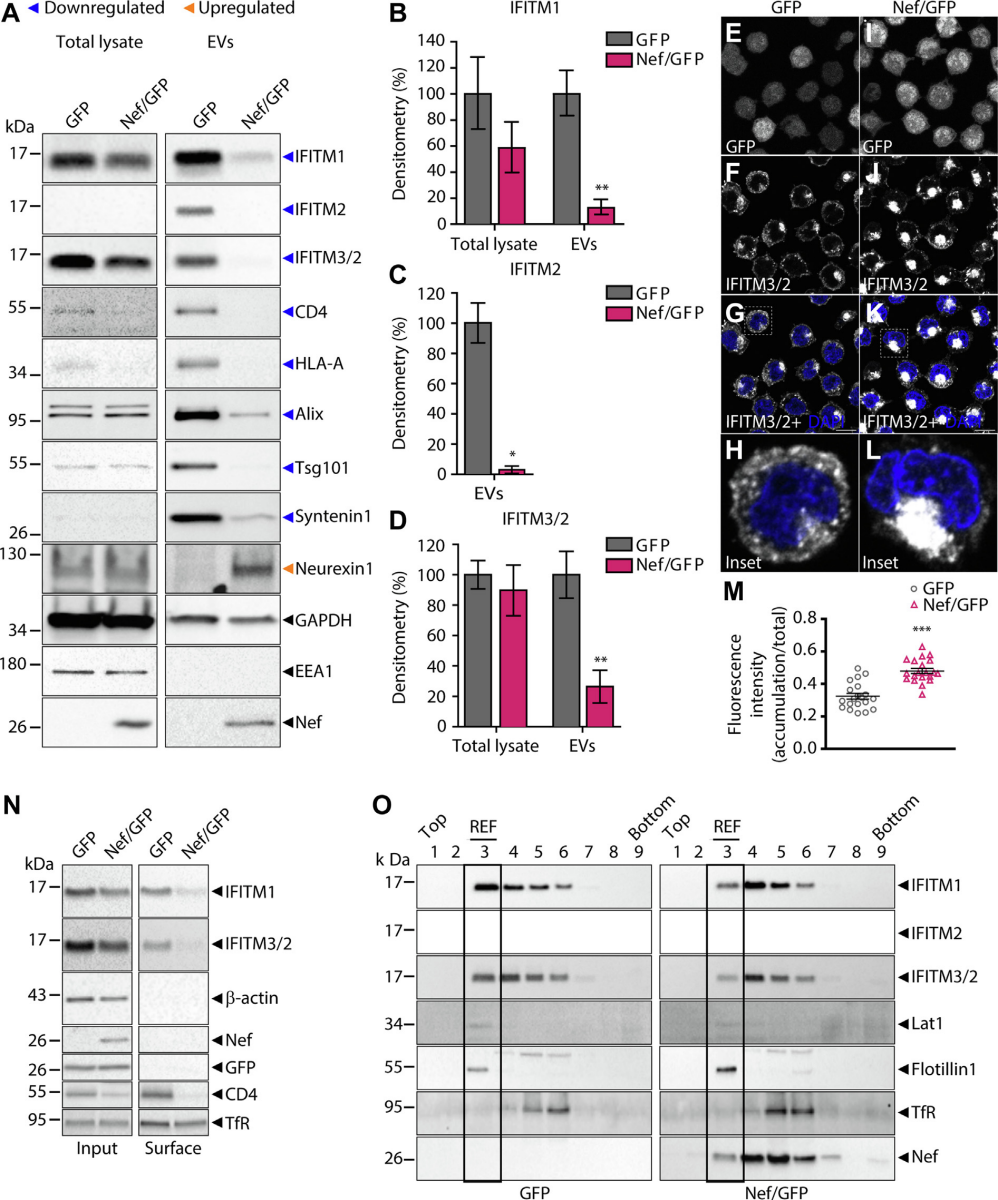
**Nef reduces the levels of typical EVs markers and host defense proteins in EVs and modifies the subcellular distribution of IFITMs.***A*, lysates from GFP or Nef/GFP T cells and their respective NTA equalized EVs were subjected to SDS-PAGE and Western blot with antibodies against indicated proteins. Nef strongly reduces the levels in EVs (*Blue arrowheads*) of known Nef targets (CD4 and HLA-A), some typical EVs markers (Alix, Tsg101, and syntenin1), and proteins with known anti-HIV activity (IFITM1, IFITM2, and IFITM3). In contrast, the EV levels of neurexin1 were highly increased by Nef. GAPDH abundance is not altered in EVs. EEA1 was used as a control for the cytosolic contaminant. The levels of IFITM2 were below the detection limit in total cell lysates. Representative Western blot from three independent experiments. *B*–*D*, band densitometry graphs (%), of total lysate and EVs, of IFITM1 (*B*), IFITM2 (*C*) and IFITM3 (*D*). The bar graph represents the mean ± SEM of n = 3 biological replicates. Student's *t* test; ∗*p* < 0.05; ∗∗*p* < 0.01. *E*–*H*, GFP and (*I*–*L*) Nef/GFP cells were fixed, permeabilized and immunolabeled to endogenous IFITM3/2. The cells were analyzed under confocal microscope. Nef modifies the subcellular distribution of IFITM3. The bars represent10 μm. *M*, values represent the ratio (accumulation/total) of fluorescence intensity mean ± SEM from 20 different cells for each condition. Student's *t* test; ∗∗∗*p* < 0.001. *N*, GFP and Nef/GFP T cells were surface biotinylated. The biotinylated surface proteins (surface) were probed with the indicated antibodies. Input represents 1% of total cell lysate. Nef reduces the IFITM1 and IFITM3/2 levels from the cell surface. Representative Western blot from two independent experiments. *O*, the total lysates of GFP and Nef/GFP T cells were ultracentrifuged onto a sucrose gradient to separate fractions enriched or not in Flotillin1 positive lipid rafts. A total of nine fractions were collected from the top of the gradient. In Nef/GFP T cells, IFITM1, 2, and 3 had their levels reduced in lipid rafts enriched fraction (REF, fraction 3), defined by the presence of lipid rafts markers Lat1 and Flotillin1. TfR was used as a non-raft marker. EV, extracellular vesicles; IFITM, interferon-induced transmembrane; NTA, nanoparticle tracking analysis; TfR, transferrin receptor.

**Supplemental Figure 3:**
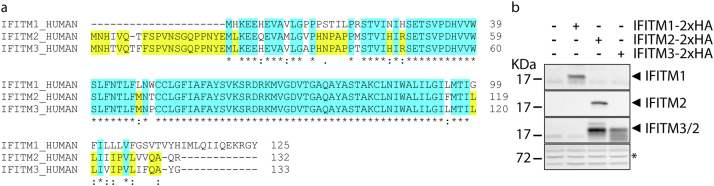


Sequence alignment between IFITM family members with characterized antiviral activity showed a similarity of approximately 82% among IFITM1, IFITM2, and IFITM3, while IFITM2 and IFITM3 are 91% similar (supplemental Fig. S3*A*). Therefore, the effect of Nef on IFITM1, IFITM2, and IFITM3 levels was analyzed. To this end, the specificity of commercial antibodies was tested in lysates of HEK293T cells transfected with plasmids encoding IFITM1-2xHA, IFITM2-2xHA, or IFITM3-2xHA (supplemental Fig. S3*B*). IFITM1 and IFITM2 antibodies are specific, while IFITM3 antibody also recognizes IFITM2 (supplemental Fig. S3*B*). The validation of the proteomic data was performed by Western blot analysis of the NTA-equalized EVs. Strikingly, all three IFITMs were strongly downregulated by Nef in EVs (Fig. 3, *A*–*D*). In contrast, total cellular levels of IFITMs detected with specific IFITM1 or IFITM3/2 antibodies were not significantly altered by Nef, while IFITM2 was below the detection limit in the total lysates using the specific IFITM2-antibody (Fig. 3*A*), presumably due to low expression levels.

The transmembrane proteins CD4 and HLA-A and the cytosolic EV markers Alix, Tsg101, and syntenin1 were confirmed to be reduced in EVs due to Nef expression (Fig. 3*A*). Also, as indicated by the proteomics data, the levels of neurexin1 were increased in Nef/GFP EVs, while the levels of GAPDH were similar between the EV samples (Fig. 3*A*). The purity of the EVs preparations regarding intracellular contaminants was confirmed by the absence of EEA1, a peripheral early endosomal protein (Fig. 3*A*).

### Nef Modifies the Subcellular Distribution of IFITMs and Removes These Proteins From EVs Budding Sites in T Cells

**Supplemental Figure 4:**
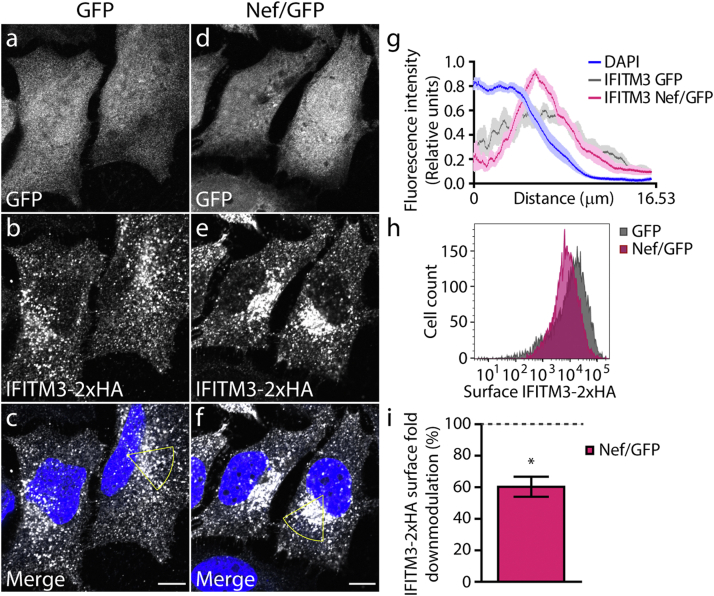


Since Nef altered the levels of IFITMs in EVs, we then investigate whether Nef changes IFITMs localization within cells. The subcellular distribution of endogenous IFITMs, detected with the IFITM3/2 antibody, was modified in lymphocytes expressing Nef and became accumulated in the juxtanuclear region compared with control conditions (Fig. 3, *E*–*M*). Similarly, exogenous HA-tagged IFITM3 (IFITM3-2xHA) coexpressed with Nef also accumulated at the juxtanuclear region in HeLa cells (supplemental Fig. S4, *A*–*G*). In both cell types, the expression of Nef leads to IFITM3 accumulation in the juxtanuclear region compared with control conditions (Fig. 3*M* and supplemental Fig. S4*G*). Because our IFITMs antibodies were not suitable for flow cytometric analysis, the surface expression of IFITMs was initially investigated in HeLa cells expressing IFITM3-2xHA. Flow cytometric analysis showed that Nef decreased by 40% IFITM3 surface levels (supplemental Fig. S4, *H* and *I*). The endogenous IFITMs surface levels in lymphocytes were analyzed by protein surface biotinylation assay. IFITM1 and . IFITM3 were downregulated at the cell surface in the presence of Nef (Fig. 3*N*). As expected, CD4 was also efficiently downregulated by Nef at the cell surface and transferrin receptor was not altered (Fig. 3*N*). Subcellular fractionation was performed to analyze IFITMs partitioning into lipid rafts (Fig. 3*O*). Nef expression led to a redistribution of IFITM1, IFITM2, and IFITM3 from lipid raft enriched fractions (REFs, fraction 3) to non-raft fractions (fractions 4–6) (Fig. 3*O*). Lipid raft enriched fractions were identified with anti-flotillin1 and anti-lat1 antibodies, two well-established lipid rafts markers (53, 54), and the non-raft fractions with transferrin receptor (Fig. 3*O*).

### Nef-Mediated Downregulation of IFITMs in T Cell EVs is Conserved in the Natural HIV-1 Nef Allele NA7

**Fig. 4:**
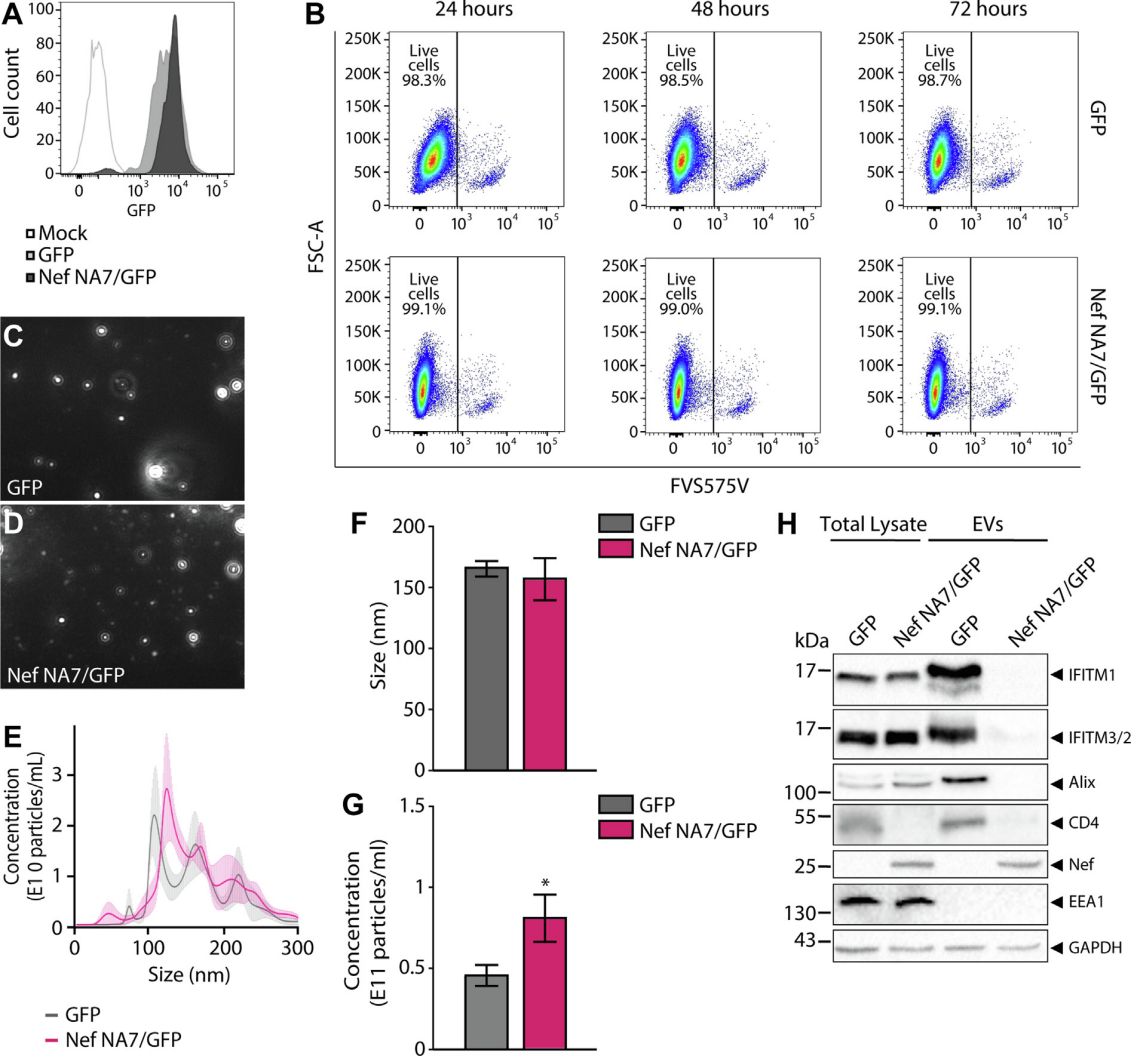
**Nef from HIV-1 NA7 variant downregulates IFITMs from EVs.** A3.01 T cells were transduced with bicistronic IRES-based retroviral vectors to express GFP (GFP) or both Nef NA7 and GFP (Nef NA7/GFP) and selected by cell sorting. *A*, histogram showing the intensity of GFP fluorescence in GFP and Nef NA7/GFP cells analyzed by flow cytometry. *B*, dot-plots of live cell population of GFP and Nef NA7/GFP cells cultivated in EVs-depleted medium for 24, 48, and 72 h at 37 °C and analyzed by flow cytometry. *C*–*G*, GFP and Nef NA7/GFP cells were incubated with an EV-depleted medium for 48 h at 37 °C, and then the supernatants were collected and the EVs were isolated by differential ultracentrifugation as described in Figure 1. *C* and *D*, representative images of the EVs from GFP (*C*) and Nef NA7/GFP (*D*) cells obtained by the NTA analysis. *E*, the size and concentration of the EVs were characterized by NTA. The line is the average value and shadow indicates ±SEM. n = 3 biological replicates, with three technical runs. *F* and *G*, bar graph represents the mean ± SEM of EVs size (*F*) and EVs concentration (*G*) analyzed by NTA. Student's *t* test; ∗*p* < 0.05. *H*, lysates from GFP or Nef NA7/GFP T cells and their respective NTA-equalized EVs were subjected to SDS-PAGE and Western blot with antibodies against indicated proteins. Nef NA7 variant strongly reduces the levels in EVs of CD4, Alix, IFITM1 and IFITM3/2. GAPDH abundance is not altered in EVs. EEA1 was used as a control for the cytosolic contaminant. EV, extracellular vesicles; IFITM, interferon-induced transmembrane; IRES, internal ribosome entry site; NTA, nanoparticle tracking analysis.

Next, we sought to confirm whether that IFITMs downregulation in T cells EVs is a conserved property of Nef. To this end, we transduced A3.01 T cells with retroviral vectors to express GFP alone (GFP) or GFP and Nef NA7 (Nef NA7/GFP), a well characterized natural HIV-1 nef allele (34), and selected GFP-positive cells by cell sorting (Fig. 4*A*). As described above for Nef NL4/GFP cells, viability was analyzed after 24, 48, and 72 h of EVs production and no impairment in the cell viability was observed (Fig. 4*B*). Culture supernatants of Nef NA7/GFP and GFP cells conditioned during 48 h were processed by differential ultracentrifugation to obtain EV-enriched fractions. NTA-based characterization of Nef NA7/GFP-EVs (Fig. 4, *C*–*F*) indicated similar features to Nef/GFP-EVs (Fig. 1, *F*–*G*), and an increased concentration of EVs released by Nef NA7/GFP cells (Fig. 4, *E* and *G*), as observed for Nef NL4-3 cells (Fig. 1, *F*–*H*). Western blot analysis of total cell and NTA-equalized EVs lysates from Nef NA7/GFP and GFP confirmed the differences in EV protein profile, validating the proteomic data (Fig. 4*H*). IFITM1 and IFITM3/2 were strongly downregulated by Nef NA7 in EVs, whereas their total cellular levels were not altered (Fig. 4*H*). The downregulation of CD4 and the cytosolic EV marker Alix in EVs, due to Nef NA7 expression, was confirmed (Fig. 4*H*), while the levels of GAPDH were similar between the EV samples (Fig. 4*H*). The enrichment of the EVs in the preparations regarding intracellular contaminants was confirmed by the absence of EEA1 (Fig. 4*H*).

### Transfer of IFITM3 to T Cells Mediated by EVs

**Fig. 5:**
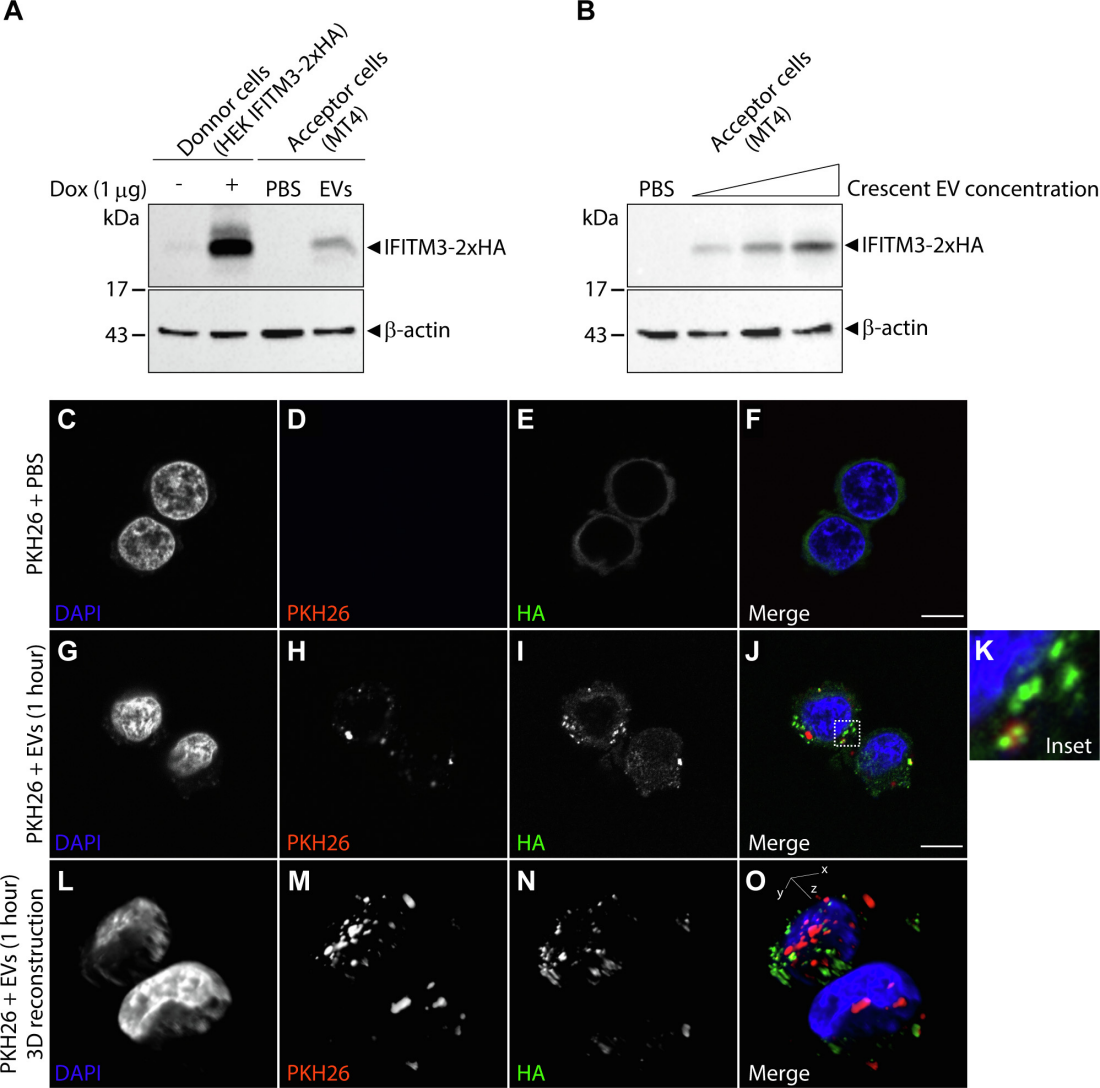
**Characterization of the IFITM3-2xHA EVs transfer to acceptor cells (MT4).***A*, EV Donor cells, HEK IFITM3-2xHA, were induced or not with doxycycline for expression of IFITM3-2xHA. For the Western blot assay, 3.0 × 10^10^ particles/ml of EVs were incubated with approximately 5.0 × 10^5^ MT4 T cells (acceptor cells) for 3 h, then the cells were extensively washed and cell lysate were prepared. IFITM3-2xHA was detected with the anti-HA antibody, demonstrating the efficiency of the transfer. *B*, 0.5; 1 and 3.0 × 10^10^ particles/ml of EVs were incubated respectively with approximately 5.0 × 10^5^ MT4 T cells (acceptor cells) for 3 h. *C*–*G*, for the immunofluorescence assay, 3.0 × 10^5^ acceptor cells were incubated with a control DPBS sample (with no vesicles, *C*–*F*) or 3.0 × 10^10^ particles/ml of EVs sample (*G*–*K*) both of which pretreated with the fluorescent reagent PKH26, as described in the Experimental Procedures. After 1 h incubation, cells were washed and immunostained with primary anti-HA antibody and Alexa-488 conjugated secondary antibody. Cells were analyzed under a confocal microscope. *L*–*O*, 3D reconstruction analysis of z-stacked confocal imagens the cells from (*G*–*J*) generated by the Fiji plugin 3D Viewer. The standard bars represent 10 μm. Representative images from two independent experiments. DPBS, Dulbecco's phosphate-buffered saline; EV, extracellular vesicles; IFITM, interferon-induced transmembrane.

**Supplemental Figure 5:**
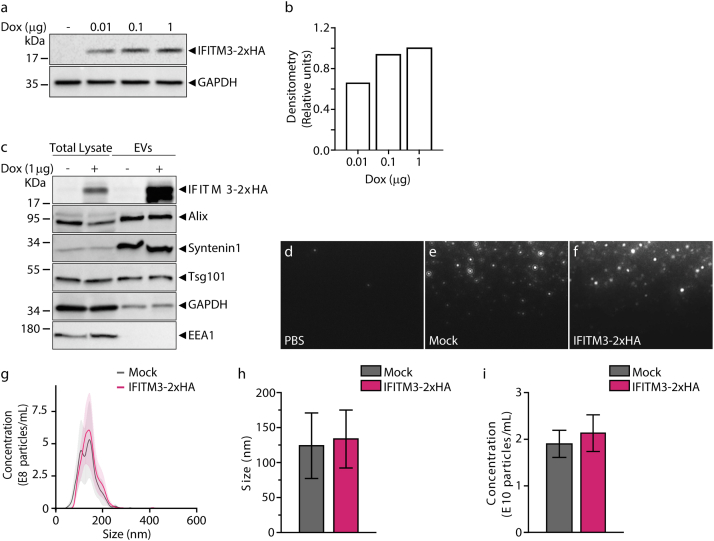


**Supplemental Figure 6:**
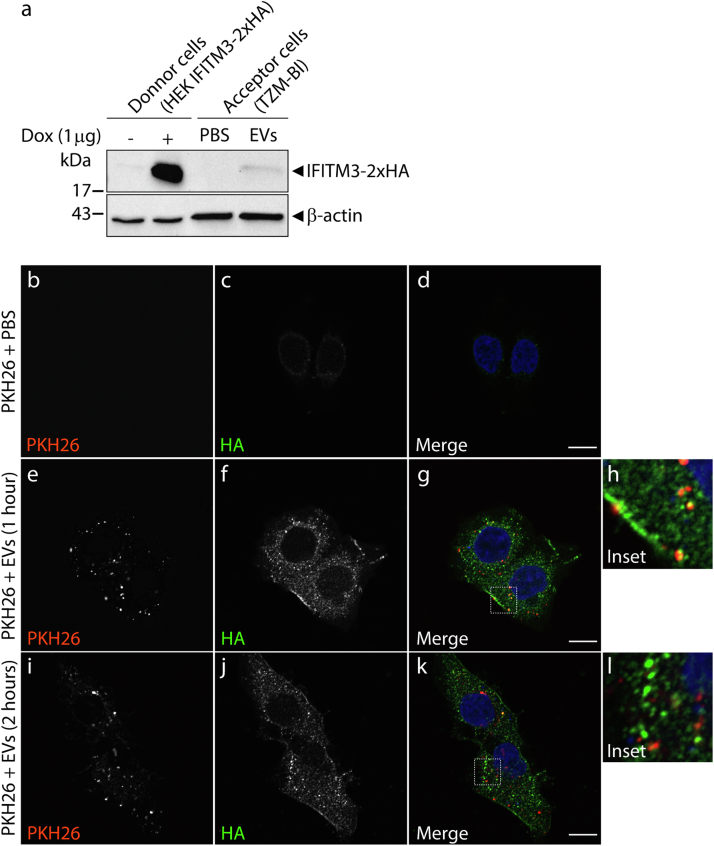


The presence of IFITM3 in T cells EVs and its depletion by Nef prompted us to test whether this host anti-HIV factor is transferable to T cells by EVs. To this end, we initially generated a HEK293 cell line expressing IFITM3-2xHA in an inducible manner (supplemental Fig. S5, *A* and *B*). Next, we purified EVs from induced HEK293-IFITM3-2xHA conditioned media and characterized the EVs by Western blot and NTA analysis (supplemental Fig. S5, *C*–*I*). As expected, the presence of IFITM3-2xHA in EVs was confirmed (supplemental Fig. S5*C*). Transference of IFITM3-2xHA from HEK293-IFITM3-2xHA EVs to MT4 T cells was confirmed by both Western blot and immunofluorescence analysis (Fig. 5, *A*–*G*). Similarly, those EVs were also able to transfer IFITM3-2xHA to TZM-bl HeLa cells (supplemental Fig. S6). Therefore, by removing IFITM3 from EVs, Nef prevents the intercellular transference of this well characterized anti-HIV factor.

## Discussion

This study establishes the HIV-1 accessory protein Nef as a global modulator of EVs proteome and identifies potential new host targets of this virulence factor. Our quantitative proteomic profiling of EVs released from a T-cell line revealed Nef-mediated changes in the EV levels of several host proteins reported to directly affect the HIV replication cycle and many other proteins not previously associated with HIV infection. Among the proteins modulated by Nef are subunits of protein complexes and/or proteins that participate in a common biological pathway. For instance, many of the proteins downregulated in EVs by Nef are known to participate in immune responses and protein trafficking, while several proteins upregulated in Nef-EVs are associated with protein synthesis and RNA stability. Interestingly, a number of proteins found in this study to be altered in EVs in response to Nef expression (supplemental Table S5) were previously shown to be modulated on the surface of HIV-1 infected T cells (55), suggesting that Nef is the viral factor modulating these surface proteins.

Specific ESCRT proteins had their levels reduced in EVs released in the presence of Nef. Among these ESCRT proteins are all four subunits of ESCRT-I (Tsg101; MVB12a and b; VPS37a, b, and c; and VPS28), all three subunits of ESCRT-II (SNF8/VPS22, VPS25, and VPS36), and Alix, an ESCRT-accessory protein. This result was somewhat surprising due to the ESCRT machinery's role in promoting EVs biogenesis (1). ESCRT-I, ESCRT-II, and Alix play essential roles in recruiting cargoes to EVs, perhaps explaining why EVs from Nef cells display a specific protein content, and suggesting an alternative mode of cargo selection for EVs in Nef cells. Of note, ESCRT machinery components directly involved in membrane deformation and scission, namely ESCRT-III subunits (CHMP4a, CHMP4b, CHMP6, and CHMP1a) and VPS4b (56), were not significantly changed or were slightly increased in Nef EVs.

Several aminoacyl-tRNA synthetases (ARSs) had their levels increased in Nef EVs. In addition to their well-characterized function in protein synthesis, extracellular ARSs have been implicated in other processes, including immune responses (57). Lysyl-tRNA synthetase (KARS) was shown to be released from cells into EVs, which induce proinflammatory activity in macrophages (58). Similarly, extracellular tryptophanyl-tRNA synthetase (WARS) was suggested to be involved in antiviral responses (59). Therefore, despite Nef’s ability to remove several antiviral proteins from EVs, T cells may compensate for this by loading EVs with ARSs that could act as regulators of immunity in acceptor cells.

IFITMs display well-described anti-HIV-1 activities (60), and we show here that Nef expression reduced the levels of IFITM1, IFITM2, and IFITM3 in EVs by changing their subcellular distribution. CD4 downregulation is a consistently observed characteristic among various *nef* alleles (34). Our study also demonstrates that Nef NA7, a previously characterized *nef* allele isolated from an HIV-1 infected individual (34), significantly reduces the levels of IFITMs in EVs. Therefore, antiviral IFITMs depletion from EVs likely represents a conserved function of this HIV-1 virulence factor.

We show that Nef reduces the surface levels of IFITMs and their association with lipid rafts enriched fractions, which may be relevant for different aspects of HIV infection. In lymphocytes, EV biogenesis believed to occur mainly in lipid rafts rich domains at the plasma membrane (1, 61, 62). Therefore, the exclusion of IFITMs from cell surface lipid rafts is likely Nef's mechanism to reduce the IFITMs loading into EVs.

Notably, lipid rafts are also involved in the entry process of different enveloped viruses, including HIV-1 (63). Considering that IFITMs antiviral activity is mainly associated with the blockage of viral fusion with host membranes, by removing IFITMs from HIV-1 entry sites, Nef may facilitate viral invasion *via* lipid rafts. While this activity may not be relevant for cells that are already infected, the transference of active Nef molecules to noninfected bystander cells is well documented (11, 15, 16, 21, 22, 64). Previous studies showed the intercellular transfer of biologically active IFITMs by EVs (65, 66). Interestingly, IFITMs received from EVs confers resistance to dengue virus infection to the acceptor cells (65). Our data confirmed that EVs are competent to transfer IFITM3 from donor cells to HeLa and T cells.

A study published while our manuscript was in preparation show that HIV-1 infection causes a redistribution of cargo proteins among different subpopulations of EVs released by T cells (18). Specifically, while the host MOV10 and SPN proteins were found to move away from nonviral EV subpopulations and be incorporated into virions, the host antiviral protein SERINC3 translocates away from virions and became incorporated into nonviral EVs. This relocation of SERINC3 was dependent on the presence of Nef. The study by Martin-Jaular *et al.* also revealed that Nef is integrated into a unique subpopulation of EVs, distinct from those containing other HIV proteins. They identified 32 host proteins with abundance distribution profiles across EV subpopulations closely resembling Nef's (see Table EV2, Query & nearest neighbor pred, HIV1_nef query, in 18). Significantly, our study identified 17 of these proteins as EV cargo, and all of them exhibited alterations due to Nef expression (supplemental Table S6).

Our data demonstrate the capacity of Nef alone to globally modify the protein composition of EVs released by T cells, suggesting that Nef is the viral factor responsible for at least some of the modifications observed in the context of infection (18). It is noteworthy that the presence of other HIV proteins may also influence the behavior of specific EV cargo in the context of infection, and alter the outcomes observed when Nef is expressed alone, as in this study. However, it is important to highlight that *nef* is among the earliest expressed HIV genes, leading to modifications in cellular homeostasis that favor viral replication.

Moreover, previous studies have shown that Nef possesses biological activity when transferred, in the absence of structural proteins, to uninfected cells through EVs (11, 16). Also, Nef can be present in the serum of HIV-1 infected subjects, even in viral suppression, when plasma HIV RNA levels are not detectable (67). Within this context, our findings shed light on the mechanisms through which Nef acts as an early HIV virulence factor in infected cells, as well as in uninfected bystander cells that receive EVs from Nef-expressing donor cells. These findings pave the way for further investigations into the role of Nef and EVs in HIV pathogenesis, offering new avenues for functional studies in primary T cells.

## Data Availability

All data supporting the findings of this study are available within the article. The mass spectrometry proteomics data associated with this study have been deposited to the ProteomeXchange Consortium *via* the PRIDE partner repository (68) with the dataset identifier PXD041028. The annotated spectra were deposited in MS viewer (69) with the search key: csedgbz77d.

## Supplemental data

This article contains supplemental data (18, 46, 47, 55).

## Conflict of interest

The authors declare no competing interests.

## References

[1] van Niel G., D'Angelo G., Raposo G. (2018). Shedding light on the cell biology of extracellular vesicles. Nat. Rev. Mol. Cell Biol..

[2] Couch Y., Buzàs E.I., Di Vizio D., Gho Y.S., Harrison P., Hill A.F. (2021). A brief history of nearly EV-erything - the rise and rise of extracellular vesicles. J. Extracell. Vesicles.

[3] Teow S.Y., Nordin A.C., Ali S.A., Khoo A.S. (2016). Exosomes in human immunodeficiency virus type I pathogenesis: threat or opportunity?. Adv. Virol..

[4] Dias M.V.S., Costa C.S., daSilva L.L.P. (2018). The ambiguous roles of extracellular vesicles in HIV replication and pathogenesis. Front. Microbiol..

[5] Pereira E.A., daSilva L.L. (2016). HIV-1 Nef: taking control of protein trafficking. Traffic.

[6] Buffalo C.Z., Iwamoto Y., Hurley J.H., Ren X. (2019). How HIV Nef proteins hijack membrane traffic to promote infection. J. Virol..

[7] Garcia J.V., Miller A.D. (1991). Serine phosphorylation-independent downregulation of cell-surface CD4 by nef. Nature.

[8] Schwartz O., Maréchal V., Le Gall S., Lemonnier F., Heard J.M. (1996). Endocytosis of major histocompatibility complex class I molecules is induced by the HIV-1 Nef protein. Nat. Med..

[9] Usami Y., Wu Y., Göttlinger H.G. (2015). SERINC3 and SERINC5 restrict HIV-1 infectivity and are counteracted by Nef. Nature.

[10] Rosa A., Chande A., Ziglio S., De Sanctis V., Bertorelli R., Goh S.L. (2015). HIV-1 Nef promotes infection by excluding SERINC5 from virion incorporation. Nature.

[11] Lenassi M., Cagney G., Liao M., Vaupotic T., Bartholomeeusen K., Cheng Y. (2010). HIV Nef is secreted in exosomes and triggers apoptosis in bystander CD4+ T cells. Traffic.

[12] McNamara R.P., Costantini L.M., Myers T.A., Schouest B., Maness N.J., Griffith J.D. (2018). Nef secretion into extracellular vesicles or exosomes is conserved across human and simian immunodeficiency viruses. mBio.

[13] Raymond A.D., Campbell-Sims T.C., Khan M., Lang M., Huang M.B., Bond V.C. (2011). HIV type 1 Nef is released from infected cells in CD45(+) microvesicles and is present in the plasma of HIV-infected individuals. AIDS Res. Hum. Retroviruses.

[14] Muratori C., Cavallin L.E., Krätzel K., Tinari A., De Milito A., Fais S. (2009). Massive secretion by T cells is caused by HIV Nef in infected cells and by Nef transfer to bystander cells. Cell Host Microbe.

[15] Campbell T.D., Khan M., Huang M.B., Bond V.C., Powell M.D. (2008). HIV-1 Nef protein is secreted into vesicles that can fuse with target cells and virions. Ethn. Dis..

[16] Mukhamedova N., Hoang A., Dragoljevic D., Dubrovsky L., Pushkarsky T., Low H. (2019). Exosomes containing HIV protein Nef reorganize lipid rafts potentiating inflammatory response in bystander cells. PLoS Pathog..

[17] de Carvalho J.V., de Castro R.O., da Silva E.Z., Silveira P.P., da Silva-Januario M.E., Arruda E. (2014). Nef neutralizes the ability of exosomes from CD4+ T cells to act as decoys during HIV-1 infection. PLoS One.

[18] Martin-Jaular L., Nevo N., Schessner J.P., Tkach M., Jouve M., Dingli F. (2021). Unbiased proteomic profiling of host cell extracellular vesicle composition and dynamics upon HIV-1 infection. EMBO J..

[19] Aqil M., Naqvi A.R., Mallik S., Bandyopadhyay S., Maulik U., Jameel S. (2014). The HIV Nef protein modulates cellular and exosomal miRNA profiles in human monocytic cells. J. Extracell Vesicles.

[20] Aqil M., Mallik S., Bandyopadhyay S., Maulik U., Jameel S. (2015). Transcriptomic analysis of mRNAs in human monocytic cells expressing the HIV-1 Nef protein and their exosomes. Biomed. Res. Int..

[21] Arenaccio C., Chiozzini C., Columba-Cabezas S., Manfredi F., Affabris E., Baur A. (2014). Exosomes from human immunodeficiency virus type 1 (HIV-1)-infected cells license quiescent CD4+ T lymphocytes to replicate HIV-1 through a Nef- and ADAM17-dependent mechanism. J. Virol..

[22] Lee J.H., Schierer S., Blume K., Dindorf J., Wittki S., Xiang W. (2016). HIV-Nef and ADAM17-containing plasma extracellular vesicles induce and correlate with immune pathogenesis in chronic HIV infection. EBioMedicine.

[23] Savidis G., Perreira J.M., Portmann J.M., Meraner P., Guo Z., Green S. (2016). The IFITMs inhibit Zika virus replication. Cell Rep..

[24] Brass A.L., Huang I.C., Benita Y., John S.P., Krishnan M.N., Feeley E.M. (2009). The IFITM proteins mediate cellular resistance to influenza A H1N1 virus, West Nile virus, and dengue virus. Cell.

[25] Lu J., Pan Q., Rong L., He W., Liu S.L., Liang C. (2011). The IFITM proteins inhibit HIV-1 infection. J. Virol..

[26] Compton A.A., Bruel T., Porrot F., Mallet A., Sachse M., Euvrard M. (2014). IFITM proteins incorporated into HIV-1 virions impair viral fusion and spread. Cell Host Microbe.

[27] Lee W.J., Fu R.M., Liang C., Sloan R.D. (2018). IFITM proteins inhibit HIV-1 protein synthesis. Sci. Rep..

[28] Jia R., Pan Q., Ding S., Rong L., Liu S.L., Geng Y. (2012). The N-terminal region of IFITM3 modulates its antiviral activity by regulating IFITM3 cellular localization. J. Virol..

[29] Folks T., Powell D.M., Lightfoote M.M., Benn S., Martin M.A., Fauci A.S. (1986). Induction of HTLV-III/LAV from a nonvirus-producing T-cell line: implications for latency. Science.

[30] Harada S., Koyanagi Y., Yamamoto N. (1985). Infection of HTLV-III/LAV in HTLV-I-carrying cells MT-2 and MT-4 and application in a plaque assay. Science.

[31] Larder B.A., Darby G., Richman D.D. (1989). HIV with reduced sensitivity to zidovudine (AZT) isolated during prolonged therapy. Science.

[32] Takeuchi Y., McClure M.O., Pizzato M. (2008). Identification of gammaretroviruses constitutively released from cell lines used for human immunodeficiency virus research. J. Virol..

[33] Guiraldelli M.F., Berenstein E.H., Grodzki A.C., Siraganian R.P., Jamur M.C., Oliver C. (2008). The low affinity IgG receptor Fc gamma RIIB contributes to the binding of the mast cell specific antibody, mAb BGD6. Mol. Immunol..

[34] Mariani R., Skowronski J. (1993). CD4 down-regulation by nef alleles isolated from human immunodeficiency virus type 1-infected individuals. Proc. Natl. Acad. Sci. U. S. A..

[35] Chaudhuri R., Lindwasser O.W., Smith W.J., Hurley J.H., Bonifacino J.S. (2007). Downregulation of CD4 by human immunodeficiency virus type 1 Nef is dependent on clathrin and involves direct interaction of Nef with the AP2 clathrin adaptor. J. Virol..

[36] Tavares L.A., da Silva E.M., da Silva-Januário M.E., Januário Y.C., de Cavalho J.V., Czernisz É. (2017). CD4 downregulation by the HIV-1 protein Nef reveals distinct roles for the γ1 and γ2 subunits of the AP-1 complex in protein trafficking. J. Cell Sci..

[37] Tavares L.A., de Carvalho J.V., Costa C.S., Silveira R.M., de Carvalho A.N., Donadi E.A. (2020). Two functional variants of AP-1 complexes composed of either γ2 or γ1 subunits are independently required for major histocompatibility complex class I downregulation by HIV-1 Nef. J. Virol..

[38] Théry C., Witwer K.W., Aikawa E., Alcaraz M.J., Anderson J.D., Andriantsitohaina R. (2018). Minimal information for studies of extracellular vesicles 2018 (MISEV2018): a position statement of the International Society for Extracellular Vesicles and update of the MISEV2014 guidelines. J. Extracell. Vesicles.

[39] Théry C., Amigorena S., Raposo G., Clayton A. (2006). Isolation and characterization of exosomes from cell culture supernatants and biological fluids. Curr. Protoc. Cell Biol..

[40] Rappsilber J., Mann M., Ishihama Y. (2007). Protocol for micro-purification, enrichment, pre-fractionation and storage of peptides for proteomics using StageTips. Nat. Protoc..

[41] Villén J., Gygi S.P. (2008). The SCX/IMAC enrichment approach for global phosphorylation analysis by mass spectrometry. Nat. Protoc..

[42] Cox J., Neuhauser N., Michalski A., Scheltema R.A., Olsen J.V., Mann M. (2011). Andromeda: a peptide search engine integrated into the MaxQuant environment. J. Proteome Res..

[43] Cox J., Mann M. (2008). MaxQuant enables high peptide identification rates, individualized p.p.b.-range mass accuracies and proteome-wide protein quantification. Nat. Biotechnol..

[44] Tyanova S., Temu T., Sinitcyn P., Carlson A., Hein M.Y., Geiger T. (2016). The Perseus computational platform for comprehensive analysis of (prote)omics data. Nat. Methods.

[45] Kalra H., Simpson R.J., Ji H., Aikawa E., Altevogt P., Askenase P. (2012). Vesiclepedia: a compendium for extracellular vesicles with continuous community annotation. PLoS Biol..

[46] Pathan M., Keerthikumar S., Ang C.S., Gangoda L., Quek C.Y., Williamson N.A. (2015). FunRich: an open access standalone functional enrichment and interaction network analysis tool. Proteomics.

[47] Pathan M., Keerthikumar S., Chisanga D., Alessandro R., Ang C.S., Askenase P. (2017). A novel community driven software for functional enrichment analysis of extracellular vesicles data. J. Extracell. Vesicles.

[48] Chen E.Y., Tan C.M., Kou Y., Duan Q., Wang Z., Meirelles G.V. (2013). Enrichr: interactive and collaborative HTML5 gene list enrichment analysis tool. BMC Bioinformatics.

[49] Kuleshov M.V., Jones M.R., Rouillard A.D., Fernandez N.F., Duan Q., Wang Z. (2016). Enrichr: a comprehensive gene set enrichment analysis web server 2016 update. Nucleic Acids Res..

[50] Sievers F., Wilm A., Dineen D., Gibson T.J., Karplus K., Li W. (2011). Fast, scalable generation of high-quality protein multiple sequence alignments using Clustal Omega. Mol. Syst. Biol..

[51] Pužar Dominkuš P., Ferdin J., Plemenitaš A., Peterlin B.M., Lenassi M. (2017). Nef is secreted in exosomes from Nef.GFP-expressing and HIV-1-infected human astrocytes. J. Neurovirol..

[52] Brégnard C., Zamborlini A., Leduc M., Chafey P., Camoin L., Saïb A. (2013). Comparative proteomic analysis of HIV-1 particles reveals a role for Ezrin and EHD4 in the Nef-dependent increase of virus infectivity. J. Virol..

[53] Janes P.W., Ley S.C., Magee A.I. (1999). Aggregation of lipid rafts accompanies signaling via the T cell antigen receptor. J. Cell Biol..

[54] Slaughter N., Laux I., Tu X., Whitelegge J., Zhu X., Effros R. (2003). The flotillins are integral membrane proteins in lipid rafts that contain TCR-associated signaling components: implications for T-cell activation. Clin. Immunol..

[55] Matheson N.J., Sumner J., Wals K., Rapiteanu R., Weekes M.P., Vigan R. (2015). Cell surface proteomic map of HIV infection reveals antagonism of amino acid metabolism by Vpu and Nef. Cell Host Microbe.

[56] Alonso Y Adell M., Migliano S.M., Teis D. (2016). ESCRT-III and Vps4: a dynamic multipurpose tool for membrane budding and scission. FEBS J..

[57] Nie A., Sun B., Fu Z., Yu D. (2019). Roles of aminoacyl-tRNA synthetases in immune regulation and immune diseases. Cell Death Dis..

[58] Kim S.B., Kim H.R., Park M.C., Cho S., Goughnour P.C., Han D. (2017). Caspase-8 controls the secretion of inflammatory lysyl-tRNA synthetase in exosomes from cancer cells. J. Cell Biol..

[59] Lee H.C., Lee E.S., Uddin M.B., Kim T.H., Kim J.H., Chathuranga K. (2019). Released tryptophanyl-tRNA synthetase stimulates innate immune responses against viral infection. J. Virol..

[60] Shi G., Schwartz O., Compton A.A. (2017). More than meets the I: the diverse antiviral and cellular functions of interferon-induced transmembrane proteins. Retrovirology.

[61] Fang Y., Wu N., Gan X., Yan W., Morrell J.C., Gould S.J. (2007). Higher-order oligomerization targets plasma membrane proteins and HIV gag to exosomes. PLoS Biol..

[62] Booth A.M., Fang Y., Fallon J.K., Yang J.M., Hildreth J.E., Gould S.J. (2006). Exosomes and HIV Gag bud from endosome-like domains of the T cell plasma membrane. J. Cell Biol..

[63] van der Meer-Janssen Y.P., van Galen J., Batenburg J.J., Helms J.B. (2010). Lipids in host-pathogen interactions: pathogens exploit the complexity of the host cell lipidome. Prog. Lipid Res..

[64] Qiao X., He B., Chiu A., Knowles D.M., Chadburn A., Cerutti A. (2006). Human immunodeficiency virus 1 Nef suppresses CD40-dependent immunoglobulin class switching in bystander B cells. Nat. Immunol..

[65] Zhu X., He Z., Yuan J., Wen W., Huang X., Hu Y. (2015). IFITM3-containing exosome as a novel mediator for anti-viral response in dengue virus infection. Cell Microbiol..

[66] Borghesan M., Fafián-Labora J., Eleftheriadou O., Carpintero-Fernández P., Paez-Ribes M., Vizcay-Barrena G. (2019). Small extracellular vesicles are key regulators of non-cell autonomous intercellular communication in senescence via the interferon protein IFITM3. Cell Rep..

[67] Ferdin J., Goričar K., Dolžan V., Plemenitaš A., Martin J.N., Peterlin B.M. (2018). Viral protein Nef is detected in plasma of half of HIV-infected adults with undetectable plasma HIV RNA. PLoS One.

[68] Vizcaíno J.A., Côté R.G., Csordas A., Dianes J.A., Fabregat A., Foster J.M. (2013). The PRoteomics IDEntifications (PRIDE) database and associated tools: status in 2013. Nucleic Acids Res..

[69] Baker P.R., Chalkley R.J. (2014). MS-viewer: a web-based spectral viewer for proteomics results. Mol. Cell. Proteomics.

