# Supplementary material for: HIV-1 Nef Changes the Proteome of T Cells Extracellular Vesicles Depleting IFITMs and Other Antiviral Factors
Source: Mol Cell Proteomics. 2023 Nov 7;22(12):100676. doi: 10.1016/j.mcpro.2023.100676 (PMC10746527; doi:10.1016/j.mcpro.2023.100676)
Supplement: Supplemental Information [file mmc1.docx]

**Supplemental Information**

**Legends to Supplementary Figures**

**Supplementary Figure 1. Characterization of A3.01 GFP and Nef NL4-3/GFP T cells. (a)** Experimental design to obtain EVs from lymphocytes expressing either GFP or Nef NL4-3/GFP. A3.01 T cells were transduced with bicistronic IRES-based retroviral vectors to express GFP (GFP) or both Nef NL4-3 and GFP (Nef NL4-3/GFP) and selected by cell sorting. **(b)** Representative Western blot showing GFP expression in the GFP and Nef NL4-3/GFP cells and Nef expression in the Nef NL4-3/GFP cells. β-actin was used as a loading control. **(c)** GFP fluorescence intensity analysis by flow cytometry analysis. Mock are non-transduced cells. **(d)** Dot-plots of live cell population of GFP cells and Nef NL4-3/GFP cells cultivated in EVs-depleted medium for 24, 48, and 72 hours at 37 ºC and analyzed by flow cytometry assay.

**Supplementary Figure 2. Characterization of the EVs proteomic data. (a-b)** Venn diagram indicating the number of total, common and “exclusive” proteins identified for **(a)** GFP-EVs triplicates (Gray) and **(b)** Nef/GFP-EVs triplicates (Burgundy). Triplicates are numbered as (1), (2) and (3). See also Supplementary Table 1. **(c)** Pie chart indicating the percentage of proteins, identified in the EVs proteomic data, downregulated (Blue, 21.5%), upregulated (Orange, 13.7%) and Unchanged (Gray, 64.8%). **(d)** Venn diagram indicating the number of proteins in the data base Vesiclepedia (Red, 13,267 proteins) and proteins identified in the EVs proteomic data (Blue, 1130 proteins). 1,095 proteins are shared and 35 new proteins were identified in the EVs proteomic data. This analysis was done using the FunRich software 3.1.3 (Pathan et al., 2015; Pathan et al., 2017). See also Supplementary Table 3.

**Supplementary Figure 3. Characterization of the IFITMs antibodies specificity.** **(a)** Sequence alignment of the human IFITM1, IFITM2 and IFITM3. The IFITMs presented a similarity of 82% and IFITM2 and IFITM3 presented a similarity of 91%. The conserved residues between the three IFITMs are indicated in blue and conserved residues between the IFITM2 and IFITM3 are indicated in yellow. An asterisk (*) indicates positions, which have a single, fully conserved residue. A colon (:) indicates conservation between groups of strongly similar properties, scoring > 0.5 in the Gonnet PAM 250 matrix. A period (.) indicates conservation between groups of weakly similar, scoring = or < 0.5 in the Gonnet PAM 250 matrix. **(b)** The specificity of anti-IFITM antibodies was analyzed by expressing HA tagged IFITM1, IFITM2 or IFITM3 in Hek293T cells. 16 hours after transfection cells were lysed and proteins analyzed by Western blot. The anti-IFITM1 and anti-IFITM2 antibodies were specific, but the anti-IFITM3 recognizes both IFITM3 and IFITM2. Due to this unspecific recognition, we termed this antibody as anti-IFITM3/2. The (*) represents a nonspecific band detected by anti-IFITM3/2 antibody, which was used as a loading control.

**Supplementary Figure 4. Nef modifies IFITM3 subcellular distribution in HeLa cells. (a-f)** HeLa cells were co-transfected with pCINeo-IFITM3-2xHA and **(a-c)** pIRES-GFP or **(d-f)** pNef-IRES-GFP. Cells were fixed, permeabilized and immunolabeled with anti-HA antibody. The cells were analyzed under confocal microscope. Nef accumulates IFITM3 in the juxtanuclear region. A concave base triangle form (Yellow) was drawn in **(c)** and **(f)** to exemplify the fluorescence intensity measure in **(g)**. Bars, 10 μm. **(g)** The fluorescence intensity for IFITM3 accumulated into GFP cells (Gray line) and Nef/GFP cells (Burgundy line) is a function of distance from the nucleus stained with DAPI (Blue line) to cell periphery, measured using the Radial Profile plugin of ImageJ, with fixed 30 degrees integration angle and 250 radius (16.53 µm distance in radius). Were used the max intensity projection images obtained from Z-stacks. Values are the mean ± SEM of normalized fluorescence intensity from 20 cells in each group. **(h)** Histogram of flow cytometry analysis. HeLa cells co-transfected with pCINeo-IFITM3-2xHA and pIRES-GFP (Gray) or pNef-IRES-GFP (Burgundy) were used to analyze the surface levels of IFITM3 using an anti-HA antibody. **(i)** The bar graph represents the fold downmodulation in percentage of IFITM3-2xHA surface levels induced by Nef in comparison with the negative control GFP (Dashed line) from three independent experiments. Student´s t-test; * P < 0.05.

**Supplementary Figure 5. Characterization of the HEK IFITM3-2xHA cell lineage and of the EVs produced by these cells. (a)** After treatment with different concentrations of the drug doxycycline (0.01, 0.1, and 1 μg/mL), cell lysates were prepared and analyzed by Western blot. GAPDH was used as load control. The concentration of 1 μg/mL was chosen for the expression of IFITM3-2xHA in the subsequent assays. **(b)** Band densitometry graph from (a). **(c)** Protein lysates of EVs released by doxycycline (1 μg/mL) induced or uninduced HEK IFITM3-2xHA cells and respective total lysate compared by Western blot. IFITM3-2xHA was efficiently secreted by EVs. The EEA1 marker, used as control for cellular contaminants, was not observed in the EV samples. The Alix protein, sintenin1, Tsg101, and GAPDH were used as EV markers. **(d-i)** The diameter and concentration of the vesicles were characterized by NTA. **(d-f)** Representative images of the EVs were obtained by the NTA. **(d)** PBS only. **(e)** Mock: EVs produced without doxycycline. **(f)** IFITM3-2xHA: EVs produced with doxycycline (1 μg/mL). **(g-i)** NTA analyses are from n=3 biological replicates, with 3 technical runs. The shadowing indicates ± SEM.

**Supplementary Figure 6. Characterization of the IFITM3-2xHA EVs transfer to acceptor HeLa TZM-bl cells.** After the production and enrichment of the EVs, produced with or without doxycycline (1 μg/mL), the EVs were incubated with the acceptor cells (TZM-bl) at different times. **(a)** Donor cells, HEK IFITM3-2xHA, were induced or not with doxycycline for expression of IFITM3-2xHA. For the Western blot assay, 3.0 x 10^10^ particles/mL of EVs were incubated with approximately 5.0 x 10^5^ acceptor cells for 3 hours, then the cells were washed 3 times and the cell lysate prepared. IFITM3-2xHA was detected with the anti-HA antibody, demonstrating the efficiency of the transfer. β-actin was used as a loading control. **(b-l)** For the immunofluorescence assay, approximately 1.0 x 10^5^ acceptor cells were incubated with a control DPBS sample (with no vesicles, b-d) or 3.0 x 10^10^ particles/mL of EVs sample (g-k), both of which pre-treated with the fluorescent reagent PKH26, as described in the Material and Methods. Incubation was for 1h (e-h) or 2h (b-d and i-l). Cells were washed 3 times and immunostained with an anti-HA antibody. Cells were analyzed under a confocal microscope. Bars, 10 μm. Representative images from two independent experiments.

**Legends to Supplementary Tables**

**Table S1.** List of proteins identified in the EVs derived from GFP or Nef/GFP A3.01 T cells by mass spectrometry, using label-free method in a MaxQuant plataform. For this analysis were excluded reverse sequences (16), contaminants proteins (100), and exclusive bovine protein sequences (99). For statistical analysis, we maintained the exclusive Human proteins (818), proteins shared between Human and Bovine taxonomies (345), and proteins only identified by site (52). Related to Figure 2 and Supplementary figure 2.

**Table S2.** List of differential abundant proteins given by Student's t-test (P-value < 0.05), using the label-free method in Perseus software. For this analysis, the LFQ data were log2 transformed, and the imputation of missing values was carried out for NaN, after exclusion of contaminants, reverse and exclusive bovine protein sequences. Missing values were replaced by random numbers that are drawn from a normal distribution, represent low abundance measurements. Data of adjusted p-value using Benjamini-Hochberg was reported. List of proteins in the Heat Map and Volcano plot analysis. Related to Figure 2.

**Table S3.** Comparison between vesiclepedia data and the EVs proteomic data obtained in the present study. Related to Supplementary Figure 2.

**Table S4.** List of differential abundant proteins grouped by Gene ontology biological process terms with adjusted P-values < 0.05 enriched in downregulated (Figure 2D) and upregulated (Figure 2E) proteins. Related to Figure 2.

**Table S5.** Comparison between Nef-mediated proteome modifications in EVs (this study) and cell surface (Matheson et al., 2015). Comparative analysis showing the Overlap between the list of proteins differentially abundant in Nef-positive EVs (either Downregulated or Upregulated, given by -1 < log2foldchange > 1 and by Student's t-test P-value < 0.05) identified in this study, and the list of differentially abundant proteins in the surface T cells 72 hours after HIV-1 infection. Cell surface proteins were selected for analysis after subtraction from proteins identified in the control sample (72 hours after HIV-1 infection in the presence of reverse transcriptase inhibitors, RTi) (Matheson et al., 2015) and given by the two-fold change in abundance, filtered for proteins with more than one unique identified peptide and PSM greater than three (Matheson et al., 2015, Ref 55). A total of host proteins plus Nef were identified as upregulated in both studies (analysis 1), whereas 36 host proteins were identified as downregulated in both studies. Five proteins that were upregulated in EVs were downregulated in the cell surface. Gene Ontology Biological Process information, given by EnrichR, with adjusted P-values < 0.05, are shown for proteins from each analysis result.

**Table S6.** Comparison between host proteins identified as released in the same EV subpopulation as Nef (Nef neighbors) (Martin-Jaular et al., 2021, Ref 18) and proteins modified by Nef in EVs (this study). The list of host proteins identified as “Nef neighbors” in EVs using 'neighbourhood network predictor' tool from Martin-Jaular et al., (2021), were compared against the list of EV proteins shown to be modified by Nef in this study. Information on Table EV2, Query & nearest neighbour pred, HIV1_nef query (Martin-Jaular et al., 2021), was used to obtain the list. A total 32 host proteins display abundance distribution profiles across EV subpopulations closely resembling Nef's. A total of 17 of those proteins were identified as EV cargo in the present study and all of them were shown to be altered by Nef expression.
